# Heat stress responses in a large set of winter wheat cultivars (*Triticum aestivum* L.) depend on the timing and duration of stress

**DOI:** 10.1371/journal.pone.0222639

**Published:** 2019-09-20

**Authors:** Krisztina Balla, Ildikó Karsai, Péter Bónis, Tibor Kiss, Zita Berki, Ádám Horváth, Marianna Mayer, Szilvia Bencze, Ottó Veisz

**Affiliations:** 1 Molecular Breeding Department, Agricultural Institute, Centre for Agricultural Research, Hungarian Academy of Sciences, Martonvásár, Hungary; 2 Crop Production Department, Agricultural Institute, Centre for Agricultural Research, Hungarian Academy of Sciences, Martonvásár, Hungary; 3 Cereal Breeding Department, Agricultural Institute, Centre for Agricultural Research, Hungarian Academy of Sciences, Martonvásár, Hungary; 4 Research Institute of Organic Agriculture, Budapest, Hungary; Institute of Genetics and Developmental Biology Chinese Academy of Sciences, CHINA

## Abstract

The adverse effects of heat on plant yield strongly depend on its duration and the phenological stage of the crops when the heat occurs. To clarify the effects of these two aspects of heat stress, systematic research was conducted under controlled conditions on 101 wheat cultivars of various geographic origin. Different durations of heat stress (5, 10 and 15 days) were applied starting from three developmental stages (ZD49: booting stage, ZD59: heading, ZD72: 6^th^ day after heading). Various morphological, yield-related traits and physiological parameters were measured to determine the stress response patterns of the wheat genotypes under combinations of the duration and the timing of heat stress. Phenological timing significantly influenced the thousand-kernel weight and reproductive tiller number. The duration of heat stress was the most significant component in determining both seed number and seed weight, as well as the grain yield consequently, explaining 51.6% of its phenotypic variance. Irrespective of the developmental phase, the yield-related traits gradually deteriorated over time, and even a 5-day heat stress was sufficient to cause significant reductions. ZD59 was significantly more sensitive to heat than either ZD49 or ZD72. The photosynthetic activity of the flag leaf was mostly determined by heat stress duration. No significant associations were noted between physiological parameters and heat stress response as measured by grain yield. Significant differences were observed between the wheat genotypes in heat stress responses, which varied greatly with developmental phase. Based on the grain yield across developmental phases and heat stress treatments, eight major response groups of wheat genotypes could be identified, and among them, three clusters were the most heat-tolerant. These cultivars are currently included in crossing schemes, partially for the identification of the genetic determinants of heat stress response and partially for the development of new wheat varieties with better heat tolerance.

## Introduction

Global climate change is increasingly affecting crop production. Extreme weather conditions, especially temperature and rainfall anomalies, have a substantial influence on the success of cultivation. Unusually high temperature is one of the most frequent forms of abiotic stress, which represents a great danger to crop production. Extreme temperature events are expected to become more frequent in many main wheat-producing regions. These weather conditions can be characterised with short-term durations and temperature increases of over 5°C above the normal temperature [1–3]. Increasing trends can be observed in the number of heat (Tmax ≥30°C) and hot days (Tmax ≥ 35°C) [4].

The ability of wheat to adapt to a wide range of ecological conditions has made it one of the most important crops worldwide, but heat stress has severe negative effects on yield, especially when associated with other stress factors. Combined stress frequently affects wheat plants during heading or in the grain-filling period, making it essential to intensify research on the effects of heat stress [5–7]. The extent of damage is greatly influenced by the phenophase in which the plants are subjected to stress [8]. The flowering stage has generally been found to be the most sensitive to heat stress [9] because both meiosis and pollen growth are negatively affected. Complex interactions between the timing of phenological stages and the sensitivity of different growth phases to the environment influence the final yield [10].

The threshold temperature of vegetative development was reported to be 20–30°C in wheat [11], whereas that of reproductive growth was 15°C [12]. According to Tewolde et al. [13], anthesis and grain filling have a threshold temperature of 12–22°C, with significant reductions in grain yield at higher temperatures. The adverse effects of heat depend on the magnitude, timing and the duration of the stress. It was reported by Porter and Gawith [8] that in the period around flowering, the maximum temperature that wheat can endure without a decline in grain number is 31°C. This period was designated as lasting from approximately 20 days before anthesis to 10 days after anthesis [14,15]. Higher temperatures accelerate the onset of anthesis, with the consequence that there are fewer spikelets per spike [16]. In addition, high temperature near anthesis leads to reduced pollen fertility or sterile grains due to the negative effect of heat (>30°C) on pollen viability, leading to poor fertilisation, abnormal ovary development, slower pollen growth and thus a reduction in seed setting [17–20].

Wheat is often exposed to short periods of high temperature (33–40°C) during flowering and grain filling [21–23]. A 3-day period of very high temperature (max. 40°C) after anthesis was found to reduce the grain number and weight and to result in a larger number of deformed grains [24]. Even a single day of heat stress might cause serious damage to the grain yield and yield components. Rahman et al. [25] reported that high temperature led to greatly accelerated development, flowering and ripening. The grain-filling period could be 3–12 days shorter as a consequence of heat treatment [26,27]. Other authors reported that high temperatures reduced the grain-filling period by 45–60% [28,29]. However, considerable genetic variability was observed in the extent to which the grain-filling period was actually affected [30].

High temperature has a notably complex effect on numerous physiological processes, which in turn influence the photosynthetic activity of wheat plants [31,32]. Photosynthesis is one of the main metabolic processes that influence cereal yields [33], so net photosynthetic activity and chlorophyll content are important indicators of the adaptation of wheat to heat and other abiotic stress factors [34]. Both photosynthesis and dry matter yield depend on the development of optimum leaf area. Plant senescence begins with the breakdown of chlorophyll molecules, leading to retarded photosynthetic activity [35,36]. Heat stress during anthesis and grain filling were found to accelerate the degradation of the leaf chlorophyll content, resulting in a decrease in both leaf photosynthetic activity and in final biomass [31,37,38]. High temperatures also led to an increase in the rate of leaf senescence [23,39,40]. Both the grain yield and the quality are adversely affected by heat. Bhullar and Jenner [41] reported that the translocation of photosynthetic assimilates during grain filling was negatively affected by heat stress, resulting in a decline in grain quality. Heat stress causes tissue dehydration and poorer CO2 assimilation during the reproductive stage [42]. The uptake of CO2 from the air is influenced by stomatal closure and opening, so the dependence of this process on temperature is of great importance. The enhanced transpiration caused by high temperature induces stomatal closure, which has an indirect effect on the fixation of CO2 in the course of photosynthesis. The inhibition of photosystem II (PSII), the most thermally labile component of the photosynthetic electron transport chain, might be responsible for the retardation of photosynthesis, the disruption of electron transport activity and the inactivation of the oxygen-evolving enzymes of PSII, which lead to lower rates of ribulose-1,5-bisphosphate (RuBP) regeneration [30,43–46].

To further improve abiotic stress tolerance, it is highly important to evaluate the diversity in the stress reaction types of cultivated wheat varieties and to identify genotypes with higher levels of stress tolerance. A strong need also exists to identify and characterise the various mechanisms involved in tolerance and to identify the genetic components underlying these mechanisms. However, most studies use only a limited number of wheat genotypes [19,47,48], and notably little research has systematically compared the effects of heat stress of various durations when applied in various developmental phases.

Because assessment of heat tolerance is an important component in breeding programmes aimed at improving the ecological adaptation of cereals, research on heat stress tolerance has begun in the Agricultural Institute (MTA ATK MGI), in Martonvásár [49–51]. To identify the various types of stress responses in different wheat cultivars, experiments were conducted under controlled growth conditions, making it possible to use the same experimental setup across the different experiments and to apply heat stress at exactly the same phenological stage in each wheat cultivar, making the comparisons more precise.

Based on previous studies, a systematic research was planned, including a large set of wheat cultivars with wide genetic background (i) to evaluate the effect of various durations of heat stress in different plant developmental phases on physiological and yield-related traits and (ii) to apply detailed phenotypic characterisation under various heat stress treatments, making it possible (iii) to analyse the heat-stress dependent associations between the various components in forming grain yield and (iv) to identify whether specific clustering of wheat genotypes can be found based on their heat stress response profiles, as measured by the changes in grain yield /plant. This information will make it possible to initiate crosses between the various members of the identified heat stress response clusters both for breeding purposes and for evaluation of the genetic components of heat stress tolerance.

## Material and methods

### Crop management

A total of 101 winter wheat varieties with different geographic origins (S1 Table) were included in a series of experiments performed under controlled conditions in the greenhouse and phytotron to study their responses to various durations of heat stress applied at different developmental stages. The heat stress responses of the wheat varieties were determined in three independent experiments in which the same standard plant raising protocols were applied. One experiment covered screening of heat stress response in one phenological phase, and the three developmental phases examined were the booting stage (ZD49), the heading stage (ZD59) when the emergence of the inflorescences was complete, and the 6^th^ day after heading (ZD72). Heat stress treatment lasted for 5 (H5), 10 (H10) or 15 (H15) days in all three developmental stages. The phenophases of the plants were monitored every day and were determined based on the double-digit system of the Zadoks scale [52]. As a result of monitoring the development, every wheat plant received heat stress treatment in the same specific developmental stage examined within the given experiment.

In all three experiments, the germinated seedlings were vernalised in peat blocks for 60 days at 4°C with low light intensity and short daylength, and the plantlets were transferred to individual pots holding approximately 1.5 kg of a 3:2:1 mixture of garden soil, compost and sand. The plants were raised under greenhouse conditions with daily watering and a twice-weekly supply of nutrients (Volldünger Solution, Linz, Austria, in tap-water).

### Environmental conditions

After vernalisation treatment, the plants were raised in greenhouse under a relatively standard conditions in which the ambient temperature ranged between 25°C (day) and 19°C (night), and the natural light conditions were supplemented with artificial light of 170 μmol m^–2^ s^–1^ intensity produced by metal halide lamps to reach a 16-hour photoperiod regime per 24-hour cycle. In each separate experiment of the three developmental phases, 18 plants of each genotype were raised in individual pots in the greenhouse and rotated regularly during the process of monitoring their developmental patterns. Twelve of the original 18 plants with the most similar developmental and phenological aspects were selected and included in the stress experiment, resulting in 3 plants per treatment as biological replications (C, H5, H10 and H15). The control plants of the three separate experiments represented partial technical replications.

Control plants of each variety were raised in the greenhouse throughout the lifecycle, and the planned-stress plants of each cultivar at the given phenophase were transferred to a heat stress chamber (Conviron PGV-36) in the Martonvásár phytotron for a given period of time (H5, H10 or H15). At the end of the treatment period, the plants were carried back to the greenhouse and raised with the control plants until maturity. In the heat stress chamber, the plants were kept under a 16-hour photoperiod regime and a light intensity of 350 μmol m^–2^ s^–1^ produced by metal halide lamps. The temperature profile was applied as follows: a night temperature of 20°C, a day temperature that gradually increased to 36°C and was held for 8 hours, followed by a gradual decrease of the temperature to 20°C. The relative humidity (RH%) was set to 64–68% during the day and 76% at night in the stress chamber. To calculate the vapour-pressure deficit (VPD), we applied the formula VPD = (100-RH)/100*SVP, where RH is relative humidity and SVP is saturated vapour pressure (CronkLab: http://cronklab.wikidot.com/calculation-of-vapour-pressure-deficit). Based on this calculation, the VPD in the heat stress chamber was 1.9–2.13 kPa during the day and 0.56–0.63 kPa at night. These values correspond to a hot and humid environment under heat stress [53–55]. In the greenhouse, the VPD was approximately 0.703–1.01 kPa during the day.

In total, the experimental set-up consisted of 101 genotypes × 3 plants × 3 heat stress durations × 3 developmental phases.

### Morphological measurements

Various morphological and yield parameters were measured after the plants reached harvest maturity. The morphological parameters included measurements of plant height (PH), length of the last internode (LIN), length of the main ear (EaL) and spikelet number per main ear (SPIK). The spike density (DENS) was calculated from the two latter data. The yield-related parameters were the following: number of reproductive tillers (RT), straw biomass per plant (BIOM), total above-ground biomass (straw + all ears, FBIOM), main ear weight (MEaW), main seed weight (MSW), main seed number (MSN), total side ear weight (SEAW), total side seed weight (SSW), total side seed number (SSN) and grain yield per plant (GY). The harvest index (HI), grain number per spikelet (SPS), thousand-kernel weight in the main spike (MTKW), average thousand-kernel weight (ATKW), average seed number (AS) and average seed weight (ASW) were calculated from these data.

### Physiological measurements

Among the physiological parameters, the chlorophyll content (CLR) was measured on a single occasion after heat stress treatment using a SPAD-502 instrument (Minolta, Japan), which records leaf transmittance in the red and near-infrared spectra and subsequently calculates the SPAD index from these two values. As replicates, three wheat plants (per treatments) were measured on the middle of the flag leaf for chlorophyll content.

The activity of photosynthetic properties, namely, the net assimilation rate (PN), evaporation (EVP), stomatal conductance (GS) and intercellular CO_2_ concentration (ICO) of the plants, was measured using a CIRAS 2—Portable Photosynthesis System (Tutorial version 2.03; Amesbury, MA 01913 USA). The infrared gas analysis system was equipped with a leaf cuvette that exposed 1.7 cm^2^ of leaf area. The flag leaves were kept in a leaf chamber during the measurements. External air was scrubbed of CO_2_ and mixed with a supply of pure CO_2_ to create a reference concentration of 390 μmol m^–2^ s^–1^. The CO_2_ concentration was maintained at a constant level using a CO_2_ injector with a high-pressure CO_2_ gas cartridge source. The quantum flux was set to 300 μmol m^–2^ s^–1^ and the flow rate to 200 μmol m^–2^ s^–1^. The temperature inside the leaf chamber was maintained at 22°C under control conditions and 35°C under heat stress conditions. The photosynthetic parameters were determined at the same time as the chlorophyll content. A total of 26 traits, including 5 morphological, 16 yield-related and 5 physiological traits, were examined in all treatments.

### Statistical analysis

The Statistica 6 (StatSoft Inc., Tulsa, OK, USA) and GenStat® (VSN International Ltd. 18th ed.) software packages were used in the general statistical analyses. Information on the distributions of the original data under the various treatments is represented by boxplots in S1 Fig.

The mixed linear model (REML) was used to identify the effects of the timing and duration of heat stress and of the genotypes in explaining the phenotypic variance in the measured traits. In estimating the variance components (σ^2^), all effects (genotype (G), developmental phase (D) and duration of heat (H)) were considered as random to be able to estimate the factor interactions. Principal component analysis (PCA), linear and multiple regression, and multi-variable analysis were performed on a sub-sample of 16 traits covering all three trait groups to evaluate the higher order associations among treatments, traits and genotypes.

To determine the heat stress sensitivity of the various cultivars, cluster analysis (CA) was performed on the data matrix of 101 cultivars × their grain yields (g/plant) in each of the 12 environments by applying the amalgamation rule of unweighted pair-group average within the joining tree clustering module of Statistica 6. To visualise the outcome of CA, each data point in the matrix was expressed as the magnitude of deviation from the main average of grain yield in each environment. For further dissection of the type and magnitude of interconnection among the cultivars, PCA was also conducted on the same data matrix (S2 Fig).

## Results

### Effect of timing and duration of heat stress on yield-related traits

In the experimental setup of 101 wheat genotypes × 3 developmental phases × 3 durations of heat stress, all traits were significantly influenced by the three factors but to different extents (Table 1).

**Table 1:** Variance components (%) of morphological, yield-related and physiological traits in the context of 101 wheat cultivars × three timings (developmental phase) × three durations of heat stress using the general linear model.

Traits	Genotype (G)	Dev. phase (D)	Duration of heat (H)	G×D	G×H	D×H	G×D×H	Residual
**PH**	81.9***	3.2	1.4	7.4***	0.1	1.2	3.1***	1.8*
**LIN**	53.1***	16.1*	4.7	8.6***	0.5*	4.4	5.0***	7.5***
**EaL**	74.1***	6.7	0.1	6.3***	0.6**	0.0	1.8	10.3***
**SPIK**	75.7***	0.5	0.0	11.5***	0.3*	0.0	3.5***	8.4***
**DENS**	66.7***	9.5	0.0	10.8***	0.3	0.0	3.2***	9.5***
**MEaW**	26.4***	9.1	28.8*	10.3***	1.9***	0.2	3.3***	19.9***
**MSW**	20.9***	7.7	34.3*	11.0***	2.3***	0.4	3.6***	19.8***
**MSN**	30.7***	0.1	13.4	21.7***	2.3***	4.4	8.2***	19.3***
**SPS**	21.8***	0.0	15.9	23.7***	2.1***	4.4	8.8***	23.3***
**MTKW**	19.3***	18.9*	16.1	6.8***	1.3***	10.6*	8.0***	19.0***
**RT**	28.2***	18.7*	2.5	13.3***	4.0***	8.1*	7.5***	17.8***
**SEAW**	22.3***	3.4	40.1**	2.3***	5.6***	8.5	4.9***	12.9***
**SSW**	19.7***	3.3	45.7**	2.7***	6.0***	5.9	3.5***	13.2***
**SSN**	20.9***	7.8	37.2*	4.0***	4.4***	5.8	4.0***	16.0***
**BIOM**	40.8***	9.4	8.5	19.4***	0.9***	4.5	8.5***	7.9***
**GY**	20.2***	3.1	51.6***	2.2***	4.7***	4.2	2.8**	11.2***
**FBIOM**	32.5***	4.4	30.8*	7.3***	2.5***	5.3	5.2***	11.9***
**HI**	14.6***	7.9	27.8*	16.8***	4.2***	0.5	4.8***	23.4***
**AS**	31.3***	2.0	38.6*	4.8***	3.7***	2.7	2.8***	14.2***
**ASW**	27.8***	0.1	48.8***	5.1***	4.7***	0.8	1.6	11.2***
**ATKW**	29.1***	12.4*	12.9	10.6***	3.7***	5.7	5.8***	19.8***
**EVP**	6.2***	8.4	32.9*	8.7***	10.4***	4.0	2.9**	26.5***
**GS**	4.5***	1.7	66.0***	2.3***	11.5***	1.2	1.5	11.2***
**PN**	0.7*	0.6	89.4***	0	2.1***	0.3	1.0	5.8***
**ICO**	12.9***	2.4	0.2	11.9***	22.7***	1.7	2.1*	46.1***
**CLR**	36.5***	12.5*	13.8	7.9***	2.3***	4.6	4.7***	17.8***

**PH**—Plant height, **LIN**—Last internode length, **EaL**—Main ear length, **SPIK**—Spikelet number per main ear, **DENS**—Spike density (spikelet number/cm), **MEaW**—Main ear weight, **MSW**—Main seed weight, **MSN**—Main seed number, **SPS**—Grain number per spikelet, **MTKW**—Main thousand- kernel weight, **RT**—Reproductive tillers, **SEAW**—Side ear weight, **SSW**—Side seed weight, **SSN**—Side seed number, **BIOM**—Straw biomass, **GY**—Grain yield, **FBIOM**—Total aboveground biomass (straw + all ears), **HI**—Harvest index, **AS**—Average seed number, **ASW**—Average seed weight, **ATKW**—Average thousand kernel weight, **EVP**–Evaporation, **GS**—Stomatal conductance, **PN**—Net assimilation, **ICO**—Intercellular CO_2_ concentration, **CLR**—Chlorophyll content

***, **, and * indicate differences significant at the 0.1%, 1% and 5% probability levels, respectively

**Fig 1:**
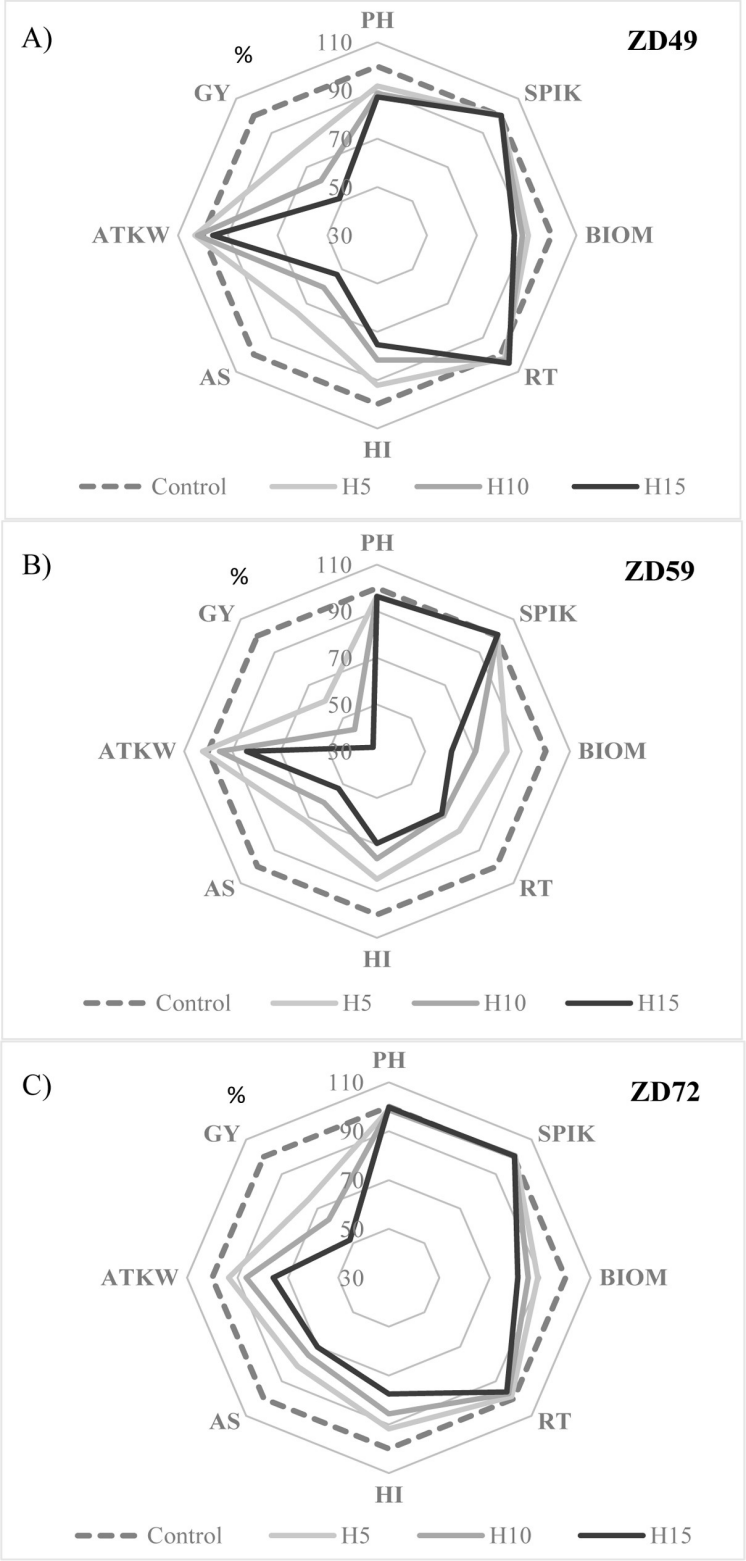
Changes in various morphological and yield-related traits in different phenophases. The values are expressed as % of the control, caused by heat stress of different durations applied in A: ZD49, B: ZD59, C: ZD72 phenophases. **PH**—Plant height, **SPIK**—Spikelet number per main ear, **BIOM**—Straw biomass, **RT—**Reproductive tillers, **HI**—Harvest index, **AS**—Average seed number, **ATKW**—Average thousand-kernel weight, **GY**—Grain yield; **H5—H10—H15**—Heat stress lasting 5, 10 and 15 days; **ZD49**—Booting stage, **ZD59**—Heading, **ZD72—**Early milk development. The confidence intervals for the traits are listed in S2 Table.

In general, morphological traits were mostly determined by the genotype, which explained between 53.1% (LIN) and 83.9% (PH) of the phenotypic variance. In the case of yield-related traits, the genotype effect was significant, but its role was smaller, explaining only between 14.6% (HI) and 40.8% (BIOM) of the phenotypic variance. In parallel, both aspects of heat stress, i.e., the developmental phase in which it was applied and especially the duration of the treatment, became more decisive factors. The developmental phase significantly influenced the thousand-kernel weight and reproductive tiller number but had no significant effect on grain yield. In contrast, the duration of heat stress was the most significant component in determining both the seed number and seed weight and was more pronounced in the case of the side ears. Via these two component groups, the duration of heat stress became the most significant component of grain yield, explaining 51.6% of the phenotypic variance (S1 Fig). Averaged over the 101 cultivars, the overall response to heat stress was a significant decrease in plant grain yield, the ratio of which worsened as the duration of the heat stress increased, regardless of the developmental phase in which heat was applied (Fig 1; for confidence intervals, see S2 Table). However, marked differences were observed between the developmental phases in terms of the extent of grain yield reduction and changes in various yield-related traits. The grain yield reduction was the largest at ZD59, when the grain yield was only 32.2% of the control value after 15-day heat stress, whereas those of ZD49 and ZD72 were similar, with 51.6 and 51.8% grain yields, respectively, compared with the control. Changes in selected yield-related traits were phenophase-specific. The reduction in AS was similarly strong at both ZD49 and ZD59, but this reduction was partially compensated by RT and ATK at ZD49, which remained stable across the treatments, and at ZD59, the reduction in AS was accompanied by strong reductions in RT and BIOM, leading to a significant decrease in ATK as the heat period lengthened. At ZD72, heat stress had no significant effect on RT, but it decreased AS, although to a lesser extent than in the other two phenophases. However, this observation was accompanied by the greatest reduction in ATKW. Heat stress had the smallest overall effect on the morphological traits, as represented by the values of PH and SPIK in Fig 1. PH decreased slightly, but this was only characteristic of the earliest developmental phase. SPIK was constant across all treatments in all three developmental stages.

### Effect of timing and duration of heat stress on photosynthesis-related parameters

In the case of physiological traits, both genotypic differences and developmental phases were less decisive, and the duration of heat stress explained the largest portion of the variance, especially for GS (66.0%) and PN (89.4%). The chlorophyll content (CLR) was the only exception, for which the genotype and the developmental phase explained 36.5% and 12.5% of the phenotypic variance, respectively. Of the interactions, both genotype × plant developmental phase and genotype × heat duration were significant variance components for most of the traits, but generally, they explained a lower portion of the variance than the main factors.

**Fig 2:**
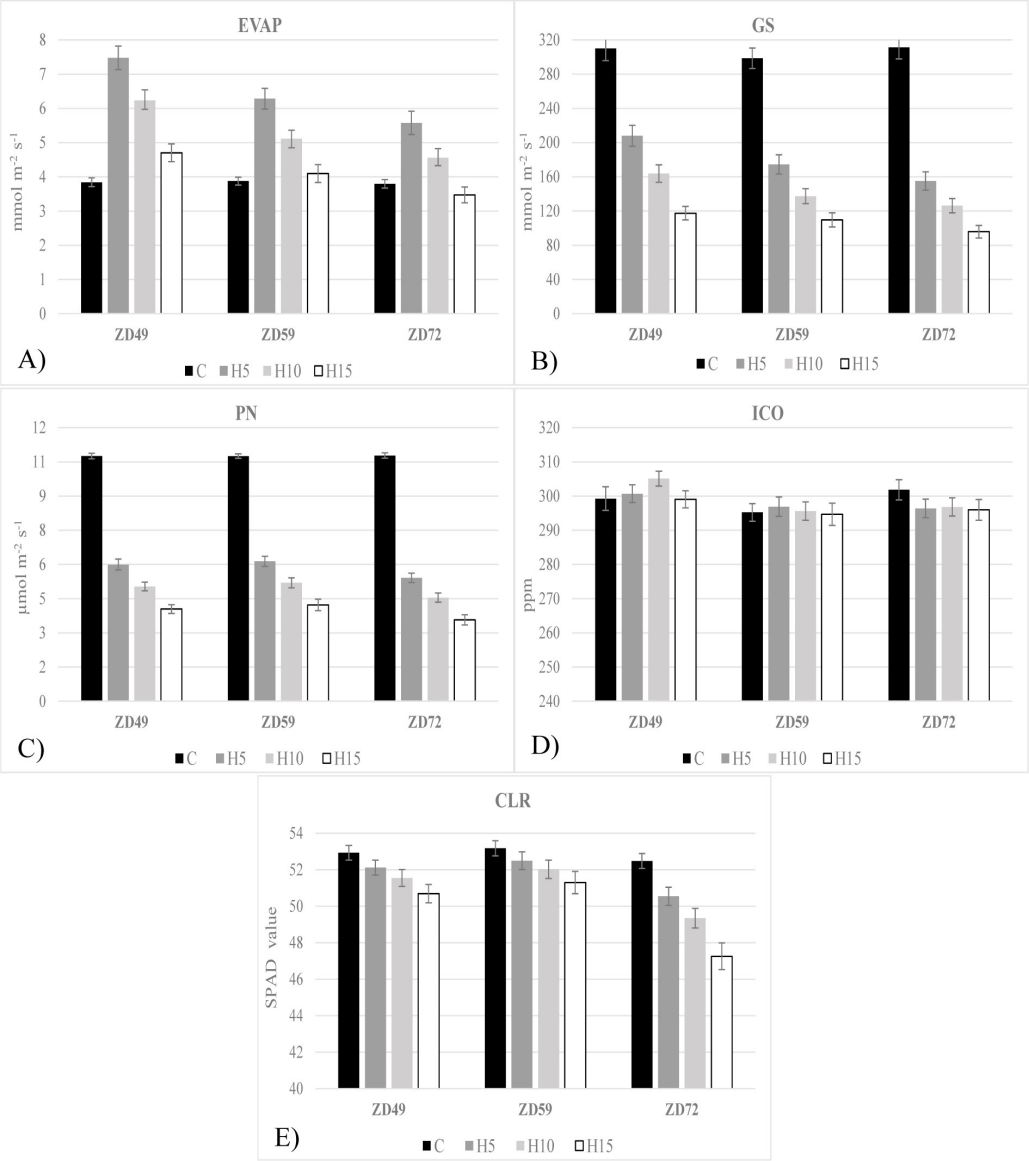
Changes in photosynthetic parameters caused by heat stress of different durations applied in different phenophases. A: ZD49, B: ZD59, C: ZD72 **EVP**—Evaporation, **GS**—Stomatal conductance, **PN**—Net assimilation, **ICO**—Intercellular CO_2_ concentration, **CLR**—Chlorophyll content, **H5**—**H10—H15—**Heat stress lasting 5, 10 and 15 days; **ZD49**—Booting stage, **ZD59**—Heading, **ZD72**—Early milk development.

In the case of physiological traits, the overall responses were similar in all three developmental phases, with differences appearing mostly across the duration of heat stress treatment (Fig 2). Stomatal conductance (GS) and net assimilation (PN) showed a strong decrease even after a 5-day heat treatment, but the values dropped only slightly in response to longer heat periods. These characteristics were somewhat intensified in later developmental phases. Interestingly, evaporation (EVP) increased to a large extent after a 5-day heat stress but subsequently gradually decreased as the heat treatment continued, decreasing to close to the control value after the 15-day heat period. For EVP, the responses were the strongest at ZD49, when it increased to almost 200% of the control after a 5-day heat period, whereas the magnitude of change lessened in later developmental phases. The values of intercellular CO_2_ (ICO) and chlorophyll (CLR) did not change significantly due to heat stress treatment at any of the phenophases.

### Heat-stress dependent associations among various components in forming grain yield

**Fig 3:**
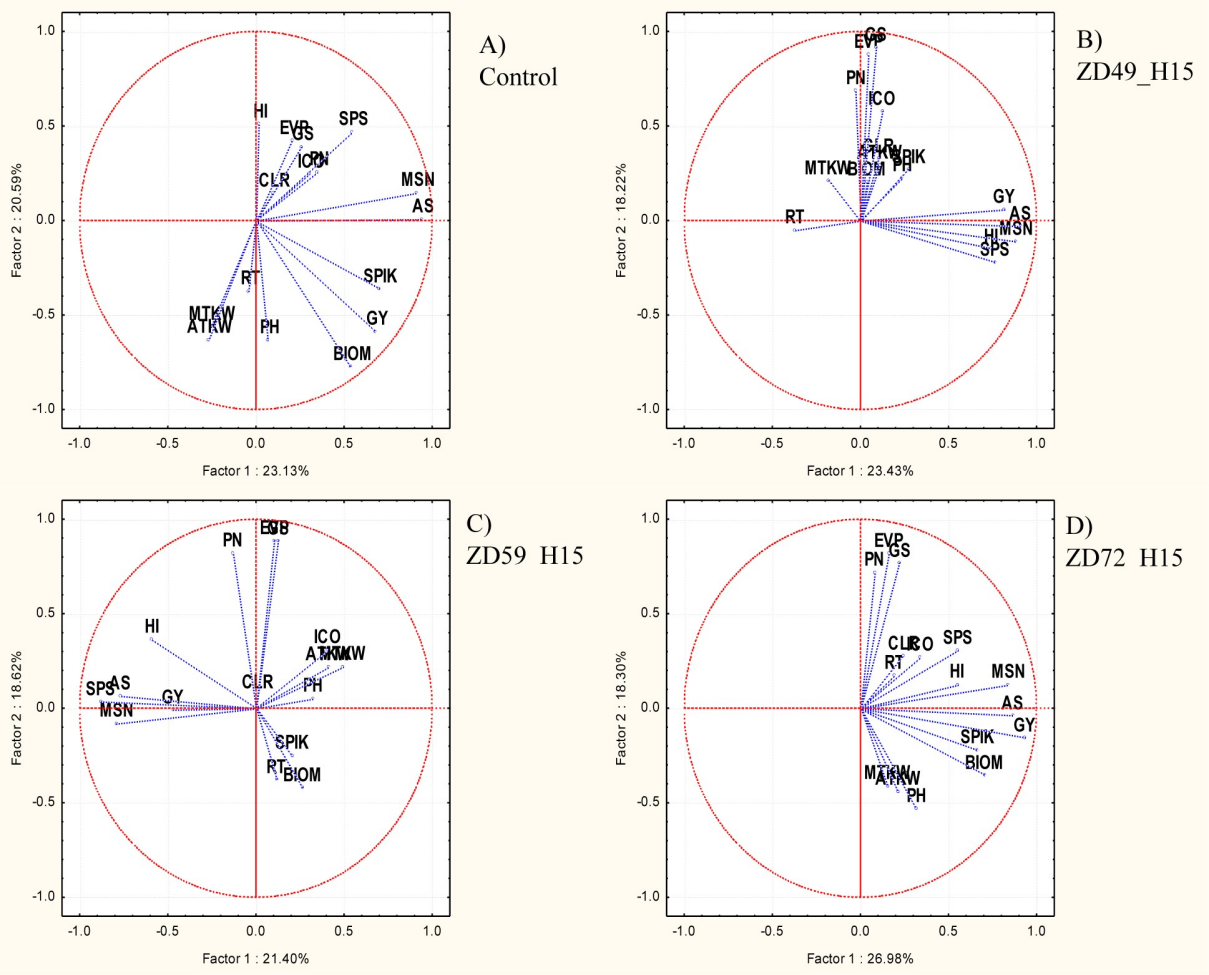
Comparison of various trait association patterns based on principal component analysis. In the Control (A) and after 15-day heat stress treatment in the phenophases ZD49 (B), ZD59 (C) and ZD72 (D). **PH**—Plant height, **SPIK**—Spikelet number per main ear, **MSN**—Main seed number, **SPS**—Grain number per spikelet, **MTKW**—Main thousand-kernel weight, **RT**—Reproductive tillers, **BIOM**—straw biomass, **GY**—Grain yield, **HI**—Harvest index, **AS**—Average seed number, **ATKW**—Average thousand-kernel weight, **EVP**—Evaporation, **GS**—Stomatal conductance, **PN**—Net assimilation, **ICO**—Intracellular CO_2_ concentration, **CLR**—Chlorophyll content.

Principal component analysis was conducted on the data matrices of the 101 cultivars × 16 traits selected to represent the three trait groups (morphological, yield-related, physiological), and the association between the traits was compared across the control and 15-day heat stress treatments in the three developmental stages (Fig 3). In the control treatment, the parameters related to seed number (AS, MSN, SPS) and thousand-kernel weight (ATKW, MTKW) formed two slightly opposing groups. Those related to seed number (AS, MSN) were more closely associated with the seed number per spikelet (SPS) and the thousand-kernel weight was more closely associated with the reproductive tiller number and plant height. The physiological characteristics grouped together, and with the harvest index and seed number per spikelet, were placed opposite to the thousand-kernel weight. Grain yield, in close association with biomass and spikelet number in the main spike, was placed intermediately to the seed number and thousand-kernel weight. In the control treatment, the biomass, seed number and grain yield were the most differentiating traits.

Phenophase-specific changes were observed in these associations after the application of 15-day heat stress, which resulted in two separate and tight groupings of the traits with the strongest differentiating powers at ZD49. One group contained the grain yield together with traits related to seed number and the harvest index, whereas the other group consisted of the physiological traits, which were most strongly influenced by evaporation and stomatal conductance. Thousand-kernel weight, biomass and reproductive tillers were only weakly associated with these two groups and had no significant effect on their formation. In the later developmental phases, heat stress did not lead to tight groupings of this type, and the associations gradually became more similar to the control as the developmental phase approached the ripening period, especially in the case of associations involving the grain yield. ZD59 still showed a tight, positive association between grain yield and seed number. Although the contribution of seed number to grain yield decreased, this observation was counteracted by the greater contribution of biomass, reproductive tiller number, and spikelet number and to a lesser extent by thousand-kernel weight and plant height. In ZD72, grain yield was grouped with the same yield-related traits as in the control, although in a tighter manner. The significance of physiological parameters in the PCA separation increased after the 15-day heat stress, irrespective of the developmental phase. However, the physiological parameters were mostly independent of the grain yield and its components in the response matrices of 101 wheat cultivars, with notably few exceptions. In the control treatment, evaporation showed a positive association with harvest index and a negative association with thousand-kernel weight. In ZD59, the intracellular CO_2_ concentration was grouped together with thousand-kernel weight and was associated negatively with grain yield to a certain extent. This type of specific association was the most pronounced in ZD72, where evaporation, net assimilation and stomatal conductance had a negative influence on thousand-kernel weight.

### Heat stress response profiles of wheat cultivars

**Fig 4:**
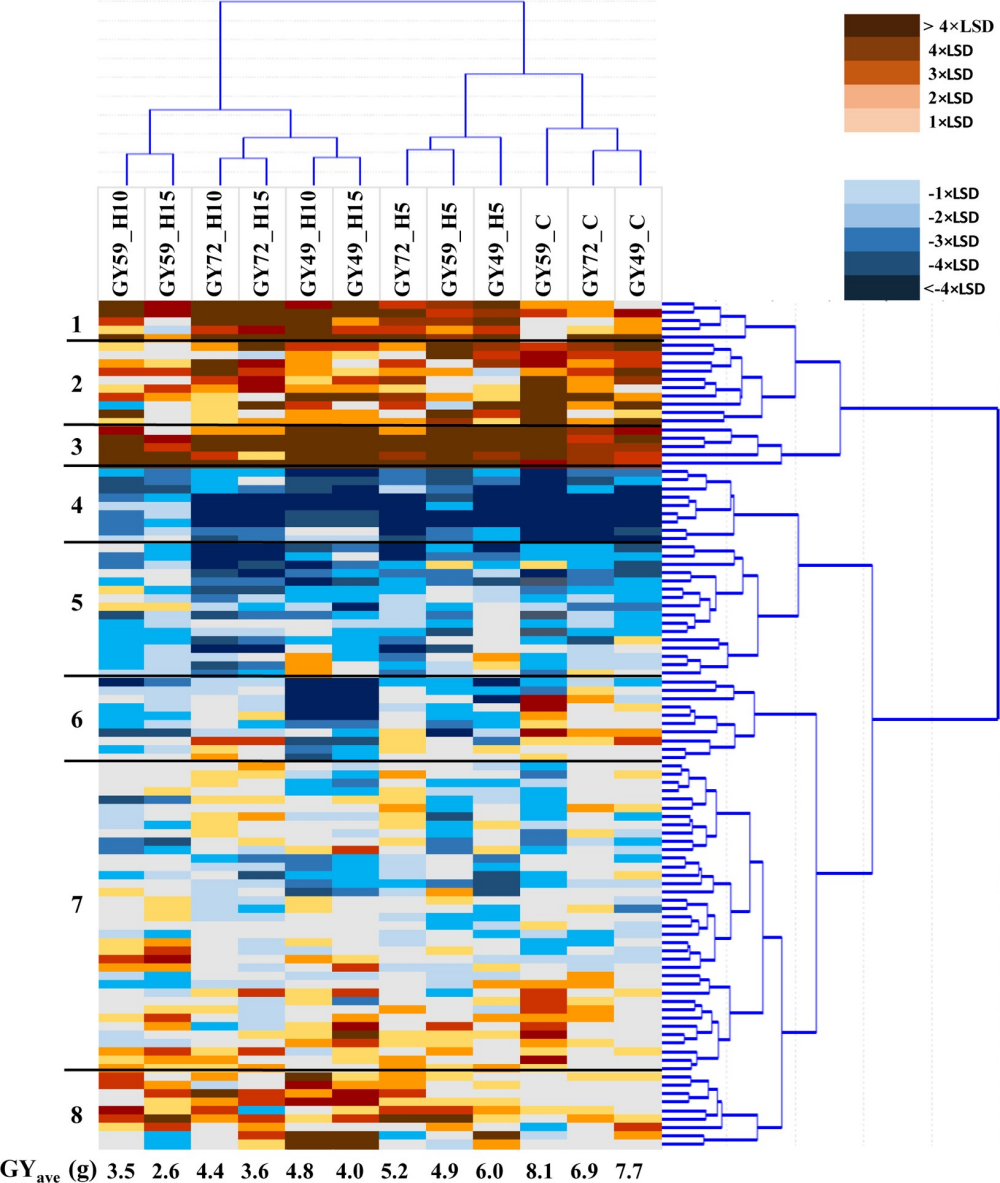
Heatmap of average grain yield/plant (g) across wheat genotypes and treatments. Rows represent 101 wheat genotypes and columns the various treatments expressed as the difference between the individual genotype and the main GY average of each treatment (within a Column). The main GY average of each treatment (g) is represented at the bottom of each column. **C**—Control, **H5—H10—H15**—Heat stress lasting 5, 10 and 15 days; **(Zadoks) 49—**Booting stage, **59**—Heading, **72**—Early milk development; **GY**—Grain yield; **LSD**—Least significant difference at P = 0.05.

Because plant grain yield is the strongest and final indicator of stress tolerance, various multi-variate analyses were conducted on the data matrix of plant grain yield in all 12 treatments × 101 genotypes to evaluate the heat-stress reactions of the wheat genotypes and establish the range of heat stress responses detectable in this wheat collection (Fig 4).

**Fig 5:**
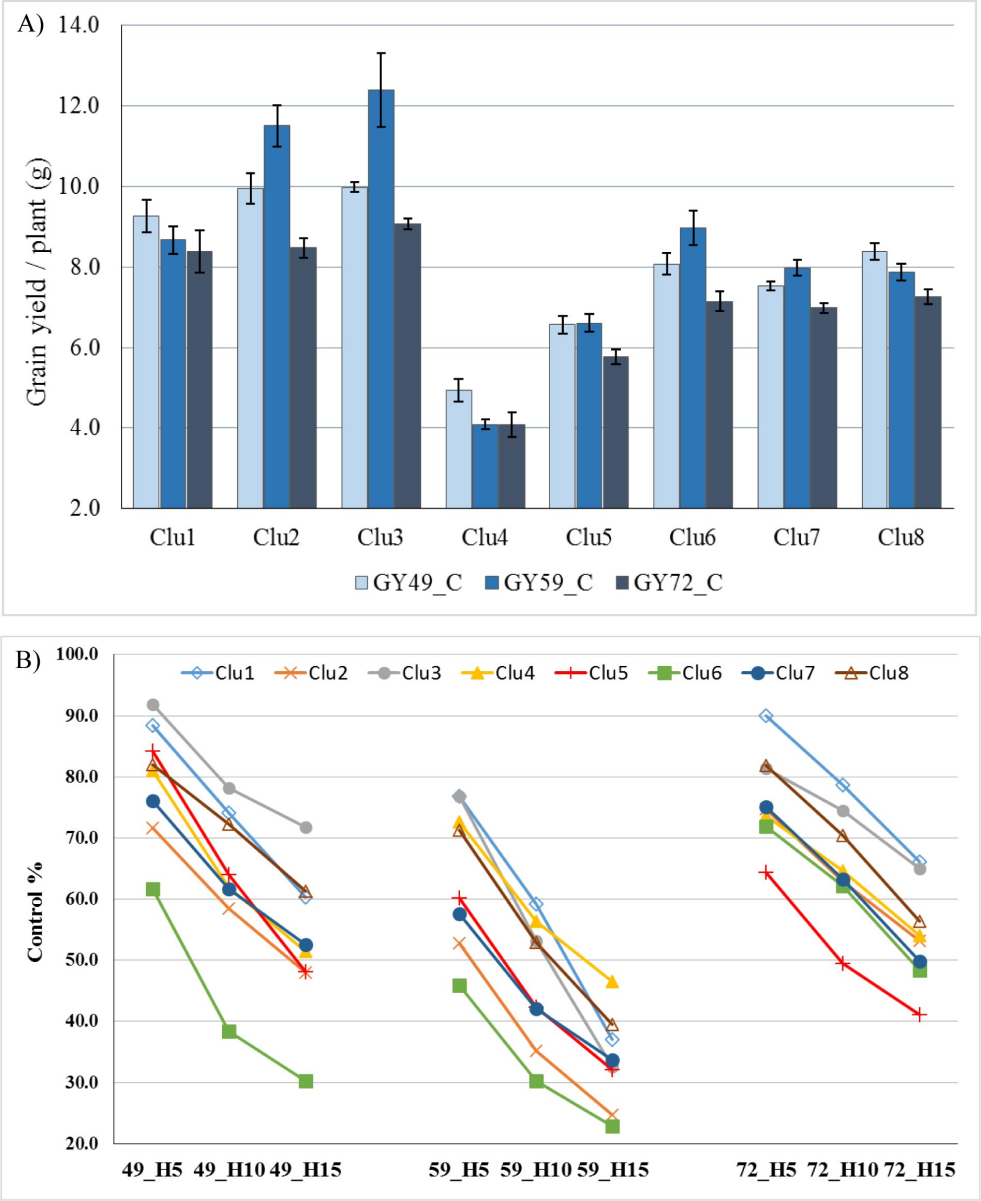
Average grain yield of the eight wheat phenotypic clusters. The clusters were identified via multi-factorial analyses. Averages values in the control treatments (A) and changes in their grain yield expressed as % of control under the various heat stress treatments (B). **C—**Control, **H5**—**H10—H15—**Heat stress lasting for 5, 10 and 15 days; **ZD49**—Booting stage, **ZD59**—Heading, **ZD72**—Early milk development.

Based on cluster analysis, eight clusters of wheat cultivars could be identified at 32% of the largest distance on the dendrogram. These clusters were also clearly separated in most cases when principal component analysis was performed on the same data matrix (S2 Fig; members of each cluster are listed in S1 Table). The only exception was Cluster 7, which was the largest group with 37 genotypes. These members showed greater dispersion along Factor 2, which correlated primarily with grain yields under longer heat stress periods at ZD59. In general, no strong association was identified between the heat stress response and geographic origin of the wheat genotypes (S1 Table), with the only exceptions of Clusters 1, 3 and 4, which had the lowest numbers of members (5, 5, and 9) but represented the extremes of yield formation. The majority of Clu1 and Clu3 were of west-European origin, whereas most of the cultivars in Clu4 came from China and Southern Europe. Based on the heat map of grain yield, Clusters 1, 2, and 3 were among the best performers across all control and heat stress treatments. The opposite was true of Cluster 4, the members of which gave the lowest grain yield regardless of treatment, followed by Cluster 5, whereas Clusters 6, 7 and 8 were intermediate in their reactions. When the grain yield of control plants was compared across the three experiments in the three developmental phases, the yielding ability of the clusters always exhibited the same order despite certain differences in magnitude between the experiments (Fig 5A).

In addition to the overall reaction patterns for grain yield, treatment-specific differences were noted between the responses of the various clusters, which could best be visualised as the average yield reduction expressed as a % of the average control values for each cluster (Fig 5B). In this way, it became evident that differences existed in the heat stress sensitivity of the individual clusters across the developmental phases, regardless of the magnitude of their yielding abilities. Of the three best-yielding clusters (Clu1, 2, and 3), the sensitivity of Clu2 was always the greatest and was more pronounced in the two earlier developmental phases. At ZD49, Clu3 proved to be the most tolerant of heat stress, whereas Clu1 gave better results at ZD72. Of the three intermediate clusters (Clu6, 7, and 8), Clu8 was the best in all three developmental phases, whereas Clu6 showed the greatest sensitivity to heat in the two earlier developmental phases (ZD49 and ZD59), not only within the intermediate clusters but also across all eight clusters. The heat sensitivity of the lowest-yielding Clu4 was intermediate for the early and late developmental phases but was among the best at ZD59. However, the heat sensitivity of Clu5 increased in later developmental phases, as a result of which this group became the most sensitive at ZD72.

With the exception of thousand-kernel weight (both in the main ear and averaged over all spikes), significant differences were observed in yield components between the clusters, both in the control and heat stress treatments (S3 Fig). The data showed that large grain number per main and average spike was the basis of high grain yield for both Clu1 and Clu3 in the control treatments, whereas a high number of reproductive tillers was the most decisive parameter for Clu2. In the control, the larger reproductive tiller number of Clu2 was able to compensate for the lower seed number, but under heat stress conditions, even the stable RT formation in both the ZD49 and ZD72 phases could not counteract the steep decrease in seed number. Under heat stress, Clu3 was better able to retain its seed number in the earlier developmental phases, especially in the main ear, whereas this ability became stronger in Clu1 in the later developmental phases. Among the intermediate clusters, the reactions of Clu6 and Clu8 are of greatest interest. The seed setting of Clu6 was the most sensitive to heat stress, leading to a severely decreased seed number in both the main ear and side spikes, which was characteristic of this group in the earlier developmental phases (ZD49, ZD59). However, the ability of Clu8 to maintain both RT and seed number was good under heat stress conditions, especially at ZD49.

## Discussion

The positive and negative aspects of experimenting under controlled and/or field-sown conditions have been previously discussed in depth by large numbers of publications [56–59]. Research has shown that results are not readily translatable from the glasshouse into the field. One of our aims in this experiment was to study the effects of heat stress in specific and well-defined developmental phases to establish how the sensitivity to heat changes with plant development. In addition, this work was performed in a larger number of wheat genotypes with different developmental patterns (the heading date window was approximately 14 days in the population). This type of systematic research is not possible to conduct under field conditions because the timing, the duration of heat or the sole stress factor can be easily controlled. This same set of wheat cultivars is a component of a long-term research programme in which the associations between plant development and yield components are planned for study under field-sown conditions for a longer time period with consideration of the various climatic factors (the results of the first three-year range have recently been published by Kiss et al. [60]). Thus, the heat stress sensitivity indices of the cultivars established in controlled conditions can later be included in temporal analyses.

The damage caused by heat stress depends on both the timing and the duration of the stress. However, most of the experiments conducted until now only consider one of these aspects, or only a limited number of genotypes are involved in the research [61–64]. In the current systematic experiment conducted with 101 wheat cultivars, both aspects of heat stress were included. In addition, the experiments were performed in a controlled environment to ensure that each genotype was exposed to heat stress in exactly the same developmental phase, thus excluding the confounding factors in field experiments, where heat stress affects the wheat genotypes in different developmental phases [62]. To study the timing of heat stress, three different developmental stages were tested, all of them after meiotic division in the male and female inflorescences. The combination of three phenophases and three heat stress periods meant that plant development took place under stress conditions for different lengths of time. When heat stress began in the booting stage (ZD49), the developmental interval under stress ranged from heading (5-day heat stress) via flowering and pollination (10-day heat stress) to seed set and early seed development (15-day heat stress). In the case of ZD59 (heading) this range stretched from flowering and pollination via seed set to early seed filling, and in the case of ZD72 (6^th^ day after heading), from seed set via early seed filling to late seed development. This scenario led to specific changes that had a stronger characteristic and significant influence, especially on yield-related traits, than the differences identified between genotypic reactions. Using this experimental setup, both aspects of heat stress proved to be highly significant determinants of various morphological, yield-related and physiological traits.

The current study confirms the results of previous research in that yield-related traits decrease with increasing heat stress duration (5, 10 and 15 days) regardless of the developmental phase when the heat stress occurs [24,49–51,65]. Five days of heat stress was sufficient to significantly decrease most yield-related traits, whereas 15-day heat stress resulted in the greatest decline. The most heat-sensitive period proved to be that following the ZD59 phenophase. The treatments that differed most from the control were ZD59_H10 and ZD59_H15, indicating that heat stress had the greatest effect on the productivity of winter wheat varieties in this stage of development. In corroboration with other studies, not only was the seed set found to be adversely affected at this stage but also the thousand-kernel weight, accompanied by a strong decrease in biomass and reproductive tiller number [22,40,63,64,66–69]. The overall grain yield reductions in ZD49 and ZD72 were observed to be similar, but this was due to specific and diverse changes in the individual yield component traits. Heat stress occurring after seed set mostly influenced the efficiency of grain filling, leading to lower thousand-kernel weight proportional to the duration of heat stress. However, in this stage, heat itself had less influence on the seed number and reproductive tiller number if water was optimally available. In contrast, when heat stress occurred before heading, the reduction in seed number was accompanied by increases in both the thousand-kernel weight and reproductive tiller number, counterbalancing the yield loss to a certain extent. This compensating effect was the most pronounced after a short period of heat and gradually disappeared as the heat duration increased.

In this study, various physiological traits related to the photosynthetic activity of the flag leaves under control and heat stress conditions were also examined to determine their roles in heat stress tolerance [32,62]. Although phenotypic diversities were noted among the wheat genotypes for all physiological parameters, this proved to be negligible compared with the effect of heat stress duration, which alone explained most of the phenotypic variations. This finding emphasises the fact that physiological changes were primarily a general response to heat stress across various genotypes. The results obtained in the current work showed that the accelerated flag-leaf senescence caused by heat stress could be attributed to lower levels of photosynthetic pigments and to a decline in photosynthetic activity.

Due to the reduced photosynthetic activity, which became more pronounced with aging of the plants, net assimilation and stomatal conductance decreased gradually with increased duration of heat stress. However, heat treatment caused a great increase in evaporation, which was more intense in younger plants, especially under the shorter period of heat stress, demonstrating that if water was available in the soil, the first general responses of plants against heat was to cool their tissues via intensified evaporation. An indirect proof of this, one observation is that genotypes with larger biomass and thus a larger area for evaporation generally tolerated heat better, as suggested by Reynolds et al. [39]. It is interesting to note that the increased evaporation occurred in spite of the strong reductions in stomatal conductance, unrelated to the phenophase. Although the stomatal conductance decreased due to heat stress, there was no complete closure, which could prevent transpiration. Sharma et al. [55] reported similar results in which the heat sensitive wheat varieties showed reduced stomatal conductance with strongly increased transpiration. In our case, most of the cultivars showed this reaction type, and the few exceptions were dispersed across all heat response clusters, underlining that no strong association exists among stomatal conductance, evaporation and heat stress tolerance in this wheat population. Because the experimental conditions represented a hot and humid environment [53–55], this phenomenon could not be caused by vapour pressure deficit. One of the possible explanations for this lack of correlation could be that the wheat cultivars studied in this work were chosen without any previous knowledge of their transpiration characteristics, and their heat stress sensitivity-tolerance has been established via grain yield reductions. This observation is in contrast to the research of Sharma et al. [55], who compared wheat genotypes that were previously selected for maximising the differences in stomatal conductance and evaporation. Among the physiological parameters, only the flag-leaf chlorophyll content showed a strong association with both genotype and developmental phase. The tendencies identified in this work were in good accordance with previous studies [29,31]. However, in the current experimental set-up, genotypic differences in chlorophyll content were not correlated with either the heat stress response or with yield-related traits, as also found by Ali et al. [61].

Thus, in the 101 wheat cultivars tested, no significant association was identified between the various parameters of photosynthetic activities and grain yield or between photosynthetic activities and heat stress tolerance. This general lack of significant associations between physiological parameters and heat stress tolerance contradicts selected data from the literature, where various parameters of photosynthetic activity were found to be associated with heat stress tolerance [55,64,70–72]. The reason for this discrepancy might lie in the different number of genotypes examined, the genetic structure of the populations, or the way in which heat stress was applied. The current work involved a larger range of wheat genotypes of different geographic origin, whereas the heat stress treatment was applied in exactly the same developmental phase for each genotype.

One of the main aims of this research was to measure the heat stress tolerance of a large set of winter wheat genotypes to characterise the extent and type of responses and identify genotypes with a higher level of heat stress tolerance. In examining the effect of developmental stage and duration of heat stress treatment, we observed that cultivars with higher grain yield under control conditions also tended to have higher grain yield under heat stress conditions. However, in spite of this observation, the yielding ability did not coincide with heat stress sensitivity expressed as the % decrease in the control grain yield. We also found that the genotypes differed in their phenophase-specific sensitivities to heat, which did not coincide with the average trends in several cases. Based on the yielding abilities across developmental phases and heat stress treatments, eight major response groups of wheat genotypes could be identified. Of these, three groups (Clu1, Clu3 and Clu8) were identified as of interest for further research. Of the two groups with high grain yield, Clu1 had the best heat stress tolerance in the early developmental phase (ZD49), whereas that of Clu3 was best in the latest developmental phase (ZD72). The heat stress tolerance of the intermediate Clu8 group was among the best in the earlier developmental phases (ZD49 and ZD59). Crosses have been initiated between the members of these three groups, partially for breeding purposes and partially for further studies to determine the genetic background of the heat stress response.

In summary, the results made it possible to describe the extent of sensitivity in different developmental phases in a larger population of wheat cultivars and to identify reaction types with increasing heat tolerance. In further research, these results could contribute to the development of varieties with better heat tolerance. It is clear that wider genetic diversity should be explored if greater heat stress resilience is to be achieved in wheat breeding programmes.

## Supporting information

S1 Table List of winter wheat cultivars included in the heat stress experiments grouped based on their heat stress response patterns, which were established with the use of cluster and principal component analyses (PCA). The position of each cultivar among and within the clusters in this table completely corresponds to its position in the heat map of grain yield (across the rows in Fig 4).
(PDF)

S2 Table 95% confidence interval of the yield-related traits in control % as a supplement to Fig 1. (PDF)

S1 Fig Boxplots of certain yield-related traits measured for 101 wheat cultivars. The values are presented in the factorial combinations of three developmental phases and three durations of heat stress.
(PDF)

S2 Fig 101 winter wheat varieties divided into eight phenotypic clusters. Clustering was carried out based on correlations of grain yield with PCA. Different coloured numbers correspond to the different heat tolerant-based groups identified via hierarchical cluster analysis.
(PDF)

S3 Fig Regression matrices of different yield-related parameters in the eight wheat clusters. (PPTX)
