# Supplementary material for: Heat stress responses in a large set of winter wheat cultivars (Triticum aestivum L.) depend on the timing and duration of stress
Source: PLoS One. 2019 Sep 20;14(9):e0222639. doi: 10.1371/journal.pone.0222639 (PMC6754161; doi:10.1371/journal.pone.0222639)
Supplement: S1 Table — The position of each cultivar among and within the clusters in this table completely corresponds to its position in the heat map of grain yield (across the rows in Fig 4). (PDF) [file pone.0222639.s001.pdf]

Cluster 1	Taxa	Pedigree	Country
	K9	DISPONENT	DE
	K194	CADENZA	GB
	K103	BONCAP	FR
	K179	MV-KOLOMPOS	HU
	K161	LUDWIG	AT
**Cluster 2**			
	K25	GK-HATTYU	HU
	K140	RECITAL	FR
	K64	FLEISCHMANN-481	HU
	K154	ZLATNA-DOLINA	HR
	K27	ROANE	US
	K142	RIGI	CH
	K155	ADRIANA	HR
	K43	LUPUS	AT
	K30	RENAN	FR
	K134	NORDIC	US
**Cluster 3**			
	K39	VANEK	DE
	K108	CUTTER	US
	K159	DUMBRAVA	RO
	K149	VALORIS	FR
	K110	ELLVIS	DE
**Cluster 4**			
	K5	MV-SUMMA	HU
	K163	NS-RANA-1	RS
	K35	AURA	RO
	K6	MV-PALOTAS	HU
	K57	FENG-YOU-3	CN
	K97	BEI-JING-0045	CN
	K190	YUMAI-34	CN
	K11	SPADA	IT
	K24	LANGFANG-3	CN
**Cluster 5**			
	K42	COURTOT	FR
	K173	MV-BERES	HU
	K96	BASTIDE	FR
	K102	BIGGAR	CA
	K107	CHARA	AU
	K74	BUCK-PANADERO	AR
	K170	SOISSONS	FR
	K120	KLEIN-FLECHA	AR
	K86	PERVITSA	RU
	K144	SAGITTARIO	IT

	K185	MV-TOBORZO	HU
	K101	BLASCO	IT
	K172	UKRAINKA	UA
	K123	KUKRI	AU
	K174	MV-BODRI	HU
	K152	WILDCAT	CA
**Cluster 6**			
	K156	BABUNA	MK
	K7	MV-MATYO	HU
	K26	MV17-09	HU
	K31	NZ4321-114	US
	K59	SAROZ	TR
	K116	KLEIN-CAPRICORNIO	AR
	K41	SALAMOUNI	US
	K122	KRASOTA	RU
	K147	SPARTACUS	GB
	K188	MV-VERBUNKOS	HU
**Cluster 7**			
	K55	BAYRAKTAR	TR
	K61	TURKMEN	TR
	K109	DIVANA	HR
	K126	MAESTRA	IT
	K78	ORNICAR	FR
	K167	SAN-PASTORE	IT
	K77	NOMADE	IT
	K176	MV-KOKARDA (MV09-09)	HU
	K85	GK-HAJNAL	HU
	K180	MV-MARSALL	HU
	K200	GERONIMO	IT
	K62	YILDIZ	TR
	K169	SKOPJANKA	MK
	K81	NW98S097	US
	K139	RAVENNA	IT
	K129	MV-AMANDA	HU
	K68	GK-BEKES	HU
	K158	DEMETRA-OS	HR
	K141	RED-RIVER-68	US
	K187	MV-KARIZMA	HU
	K143	RUZICA	HR
	K69	GK-CSILLAG	HU
	K135	NUO-MAIZI	CN
	K157	BRIANA	RO
	K13	AGENT	US
	K17	GK-HEJA	HU
	K94	BALANCE	FR

	K54	ARMCIM	TR
	K47	HALLAM	US
	K114	GK-GONCOL	HU
	K112	FERIA	AT
	K29	ORH010918	TR
	K46	GK-FENY	HU
	K184	MV-TALLER	HU
	K15	BURATINO	CZ
	K37	MV-HOMBAR	HU
	K79	MV27-07	HU
**Cluster 8**			
	K3	MV-MAGMA	HU
	K90	ARIDA	SK
	K84	GK-BERENY	HU
	K93	BALADA	CZ
	K91	ALTAY-2000	TR
	K192	KWS-SCIROCCO	DE
	K160	LIBELLULA	IT
	K171	TOMMI	DE
	K197	SUNSTAR	AU
