# Supplementary material for: Heat stress responses in a large set of winter wheat cultivars (Triticum aestivum L.) depend on the timing and duration of stress
Source: PLoS One. 2019 Sep 20;14(9):e0222639. doi: 10.1371/journal.pone.0222639 (PMC6754161; doi:10.1371/journal.pone.0222639)
Supplement: S2 Table — (PDF) [file pone.0222639.s002.pdf]

	PH	SPIK	BIOM	RT	HI	AS	ATKW	GY
**ZD49**								
C	4.42	2.36	5.46	4.19	1.47	4.07	2.49	4.02
H5	4.31	2.51	5.44	4,74	2.27	5.62	3.04	4.83
H10	4.30	2.57	5.99	5.33	2.56	6.77	2.68	5.85
H15	4.33	2.51	6.63	5.25	2.78	7.24	2.77	6.53
**ZD59**								
C	4.67	2.36	7.03	5,61	2,17	4,16	2,64	5,57
H5	4.84	2.45	8.22	5.73	5.13	5.44	2.88	6.56
H10	4.85	2.45	9.53	5.54	5.71	5.48	2.91	6.88
H15	4.87	2.55	10.18	4.95	6.25	5.38	3.30	6.13
**ZD72**								
C	4.70	2.32	5.12	4.11	1.63	4.07	2.75	4.06
H5	4.66	2.46	5.76	3.91	2.22	5.20	2.89	5.41
H10	4.69	2.31	6.01	4.48	2.38	5.51	2.88	5.92
H15	4.64	2.31	6.06	4.52	2.63	5.77	3.31	6.72

PH - Plant height, **SPIK** - Spikelet number per main ear, **BIOM** - Straw biomass, **RT** - Reproductive tillers, **HI** - Harvest index, **AS** - Average seed number, **ATKW** - Average thousand-kernel weight, **GY** - Grain yield; **C -** Control**, H5 - H10 - H15 -** Heat stress lasting 5, 10 and 15 days; **ZD49** - Booting stage, **ZD59** - Heading, **ZD72** - Early milk development
