# Supplementary material for: Heat stress responses in a large set of winter wheat cultivars (Triticum aestivum L.) depend on the timing and duration of stress
Source: PLoS One. 2019 Sep 20;14(9):e0222639. doi: 10.1371/journal.pone.0222639 (PMC6754161; doi:10.1371/journal.pone.0222639)
Supplement: S1 Fig — The values are presented in the factorial combinations of three developmental phases and three durations of heat stress. (PDF) [file pone.0222639.s003.pdf]

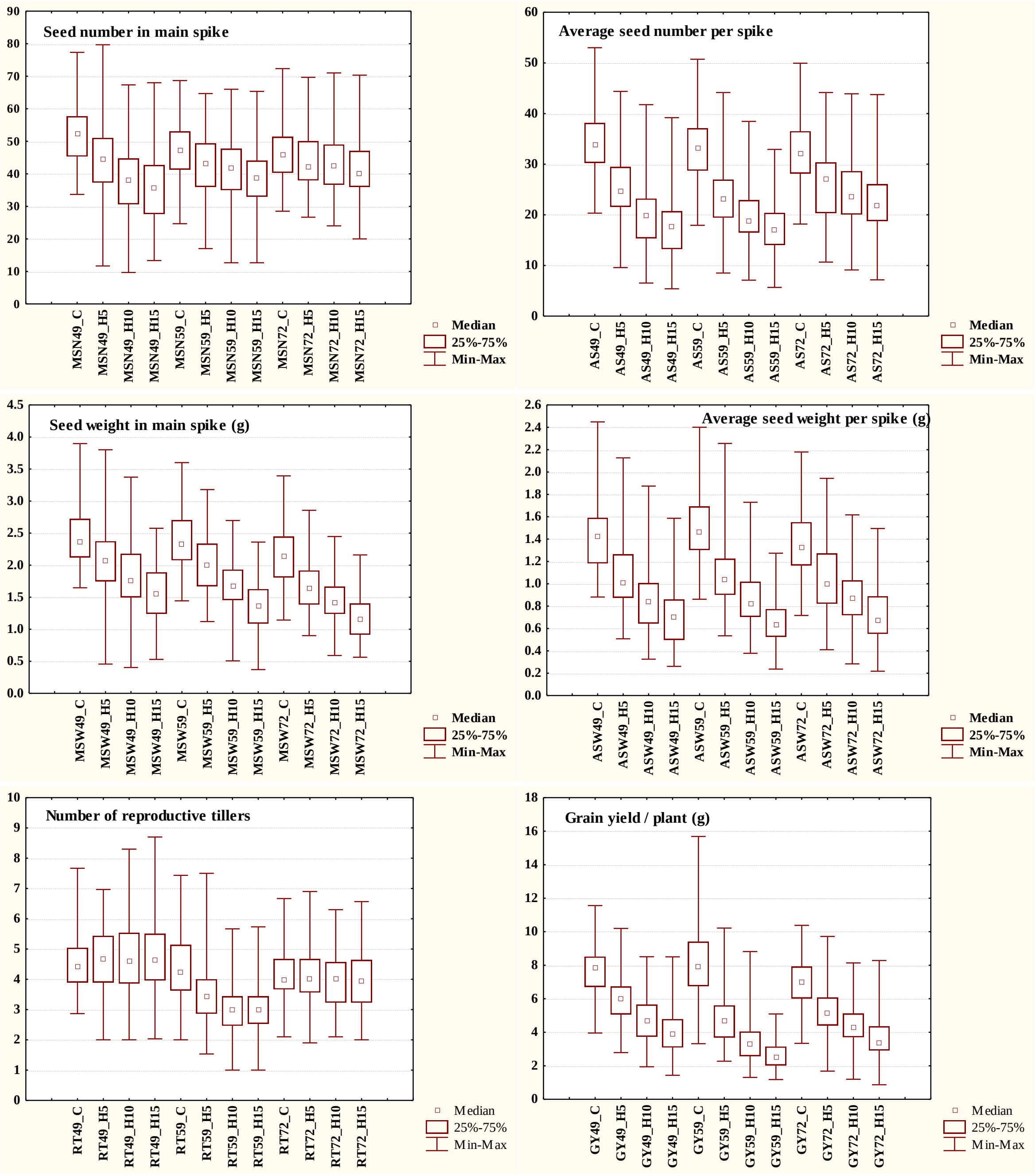

C - Control**, H5** - **H10** - **H15** - Heat stress lasting 5, 10 and 15 days; **ZD49** - Booting stage, **ZD59** - Heading, **ZD72** - Early milk development
