# Supplementary material for: Heat stress responses in a large set of winter wheat cultivars (Triticum aestivum L.) depend on the timing and duration of stress
Source: PLoS One. 2019 Sep 20;14(9):e0222639. doi: 10.1371/journal.pone.0222639 (PMC6754161; doi:10.1371/journal.pone.0222639)
Supplement: S2 Fig — Clustering was carried out based on correlations of grain yield with PCA. Different coloured numbers correspond to the different heat tolerant-based groups identified via hierarchical cluster analysis. (PDF) [file pone.0222639.s004.pdf]

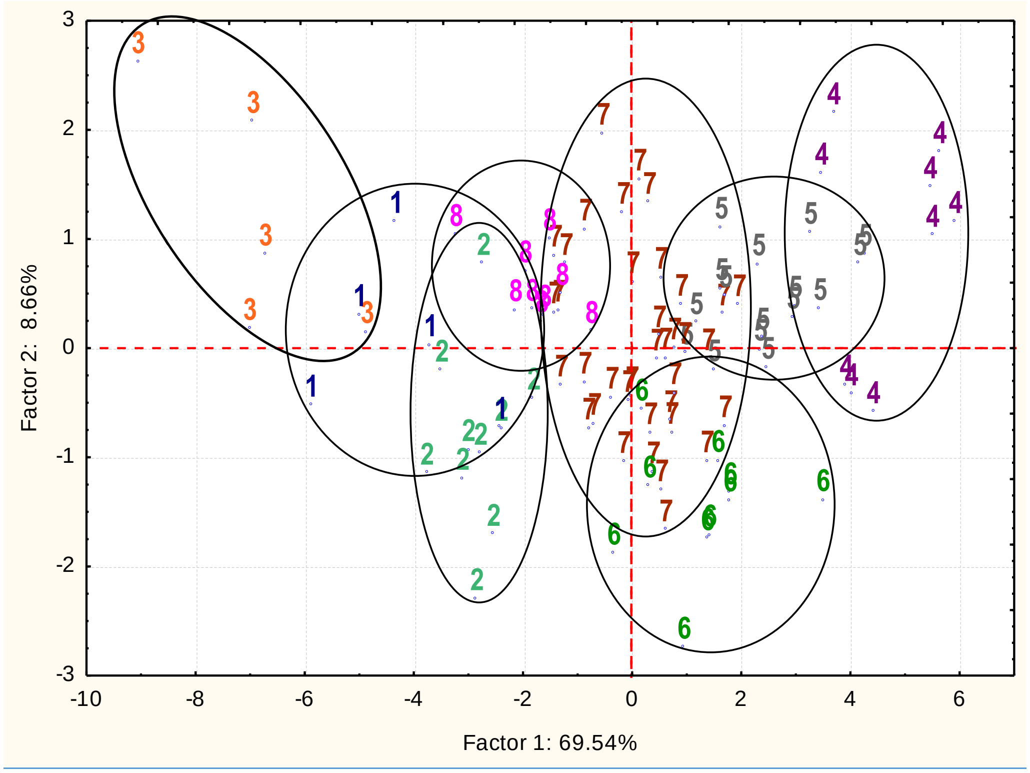

Factor – variable correlations			
Variables	Factor 1	Factor 2	Factor 3
GY49_C	**-0.826**	**-0.392**	0.031
GY49_H5	**-0.844**	0.044	**-0.383**
GY49_H10	**-0.839**	0.173	**-0.439**
GY49_H15	**-0.847**	0.259	**-0.349**
GY59_C	**-0.766**	-0.221	0.199
GY59_H5	**-0.868**	-0232	0.115
GY59_H10	**-0.833**	**0.421**	0.220
GY59_H15	-0.697	**0.511**	**0.400**
GY72_C	**-0.842**	**-0.350**	0.134
GY72_H5	**-0.886**	-0.255	0.123
GY72_H10	**-0.882**	-0.182	0.076
GY72_H15	**-0.858**	-0.157	-0.048

GY - Grain yield; **C** - Control**, H5** - **H10** - **H15** - Heat stress lasting 5, 10 and 15 days; **ZD49** - Booting stage, **ZD59** - Heading, **ZD72** - Early milk development
