# Supplementary material for: Heat stress responses in a large set of winter wheat cultivars (Triticum aestivum L.) depend on the timing and duration of stress
Source: PLoS One. 2019 Sep 20;14(9):e0222639. doi: 10.1371/journal.pone.0222639 (PMC6754161; doi:10.1371/journal.pone.0222639)
Supplement: S3 Fig — (PPTX) [file pone.0222639.s005.pptx]

## Slide 1
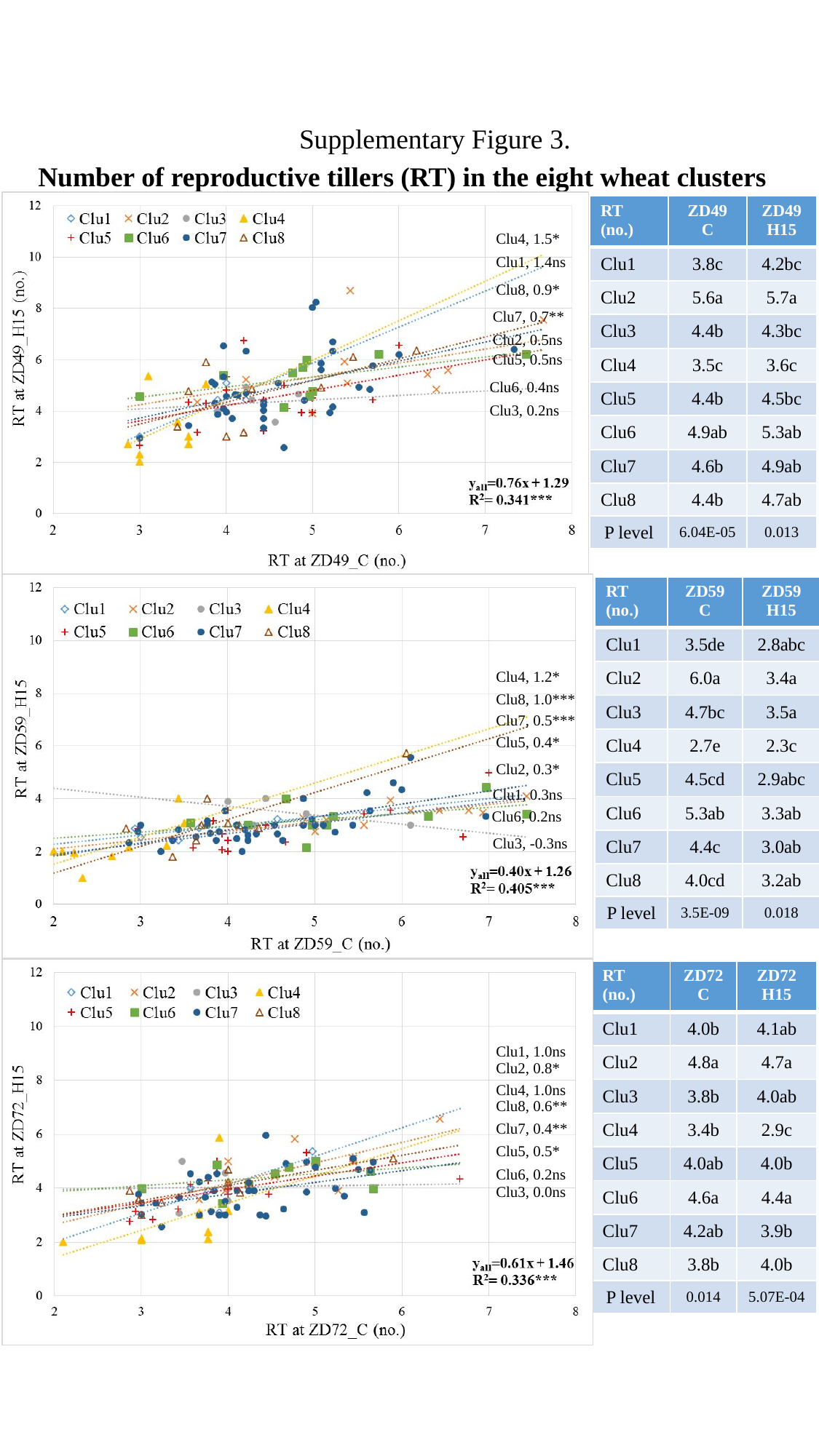

Supplementary Figure 3.
Number of reproductive tillers (RT) in the eight wheat clusters
| RT (no.) | ZD49 C | ZD49 H15 |
| --- | --- | --- |
| Clu1 | 3.8c | 4.2bc |
| Clu2 | 5.6a | 5.7a |
| Clu3 | 4.4b | 4.3bc |
| Clu4 | 3.5c | 3.6c |
| Clu5 | 4.4b | 4.5bc |
| Clu6 | 4.9ab | 5.3ab |
| Clu7 | 4.6b | 4.9ab |
| Clu8 | 4.4b | 4.7ab |
| P level | 6.04E-05 | 0.013 |
Clu4, 1.5*
Clu1, 1.4ns
Clu8, 0.9*
Clu7, 0.7**
Clu2, 0.5ns
Clu5, 0.5ns
Clu6, 0.4ns
Clu3, 0.2ns
| RT (no.) | ZD59 C | ZD59 H15 |
| --- | --- | --- |
| Clu1 | 3.5de | 2.8abc |
| Clu2 | 6.0a | 3.4a |
| Clu3 | 4.7bc | 3.5a |
| Clu4 | 2.7e | 2.3c |
| Clu5 | 4.5cd | 2.9abc |
| Clu6 | 5.3ab | 3.3ab |
| Clu7 | 4.4c | 3.0ab |
| Clu8 | 4.0cd | 3.2ab |
| P level | 3.5E-09 | 0.018 |
Clu4, 1.2*
Clu8, 1.0***
Clu7, 0.5***
Clu5, 0.4*
Clu2, 0.3*
Clu1, 0.3ns
Clu6, 0.2ns
Clu3, -0.3ns
| RT (no.) | ZD72 C | ZD72 H15 |
| --- | --- | --- |
| Clu1 | 4.0b | 4.1ab |
| Clu2 | 4.8a | 4.7a |
| Clu3 | 3.8b | 4.0ab |
| Clu4 | 3.4b | 2.9c |
| Clu5 | 4.0ab | 4.0b |
| Clu6 | 4.6a | 4.4a |
| Clu7 | 4.2ab | 3.9b |
| Clu8 | 3.8b | 4.0b |
| P level | 0.014 | 5.07E-04 |
Clu1, 1.0ns
Clu2, 0.8*
Clu4, 1.0ns
Clu8, 0.6**
Clu7, 0.4**
Clu5, 0.5*
Clu6, 0.2ns
Clu3, 0.0ns

## Slide 2
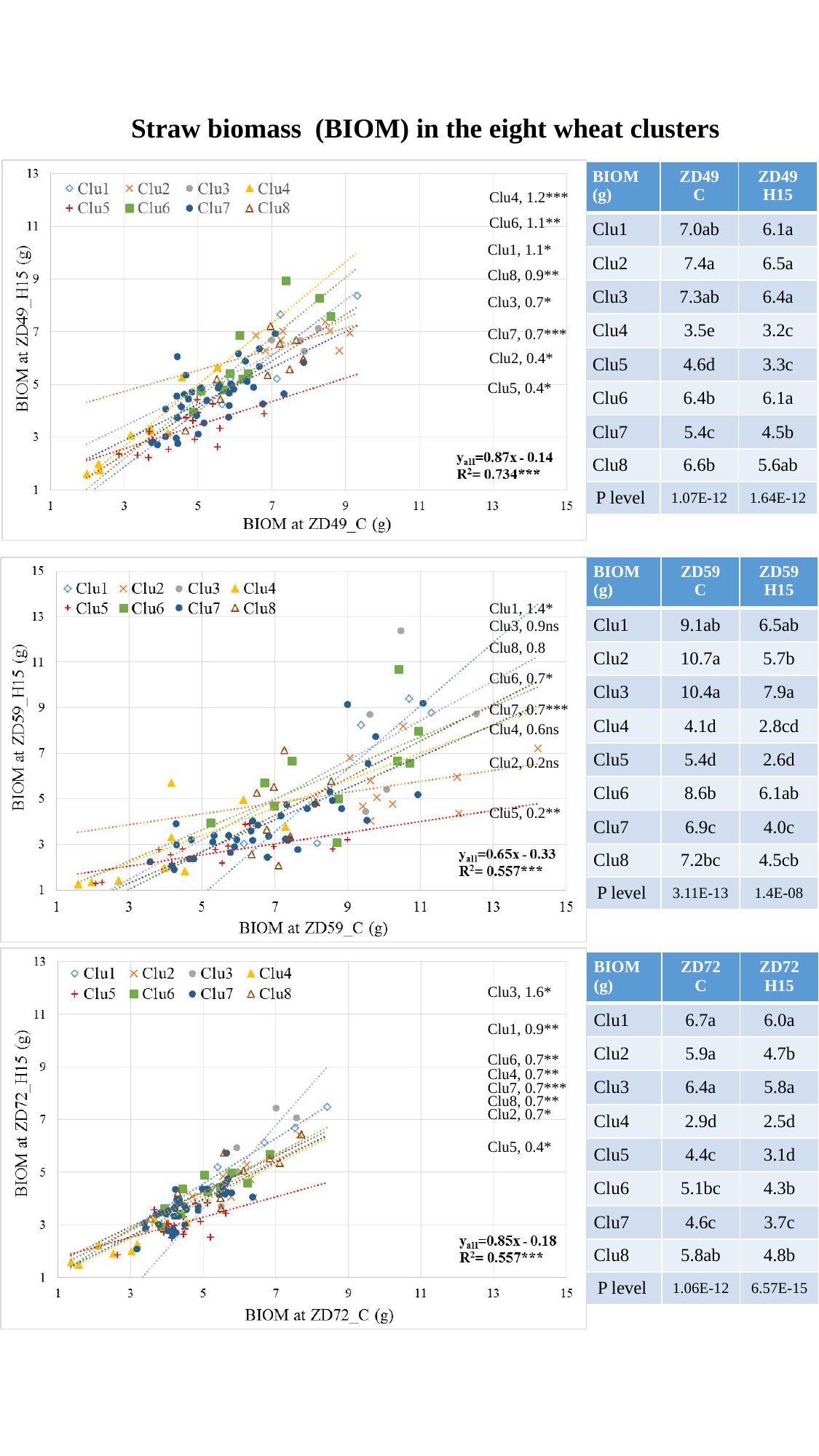

Straw biomass (BIOM) in the eight wheat clusters
| BIOM (g) | ZD49 C | ZD49 H15 |
| --- | --- | --- |
| Clu1 | 7.0ab | 6.1a |
| Clu2 | 7.4a | 6.5a |
| Clu3 | 7.3ab | 6.4a |
| Clu4 | 3.5e | 3.2c |
| Clu5 | 4.6d | 3.3c |
| Clu6 | 6.4b | 6.1a |
| Clu7 | 5.4c | 4.5b |
| Clu8 | 6.6b | 5.6ab |
| P level | 1.07E-12 | 1.64E-12 |
Clu4, 1.2***
Clu6, 1.1**
Clu1, 1.1*
Clu8, 0.9**
Clu3, 0.7*
Clu7, 0.7***
Clu2, 0.4*
Clu5, 0.4*
| BIOM (g) | ZD59 C | ZD59 H15 |
| --- | --- | --- |
| Clu1 | 9.1ab | 6.5ab |
| Clu2 | 10.7a | 5.7b |
| Clu3 | 10.4a | 7.9a |
| Clu4 | 4.1d | 2.8cd |
| Clu5 | 5.4d | 2.6d |
| Clu6 | 8.6b | 6.1ab |
| Clu7 | 6.9c | 4.0c |
| Clu8 | 7.2bc | 4.5cb |
| P level | 3.11E-13 | 1.4E-08 |
Clu1, 1.4*
Clu3, 0.9ns
Clu8, 0.8
Clu6, 0.7*
Clu7, 0.7***
Clu4, 0.6ns
Clu2, 0.2ns
Clu5, 0.2**
| BIOM (g) | ZD72 C | ZD72 H15 |
| --- | --- | --- |
| Clu1 | 6.7a | 6.0a |
| Clu2 | 5.9a | 4.7b |
| Clu3 | 6.4a | 5.8a |
| Clu4 | 2.9d | 2.5d |
| Clu5 | 4.4c | 3.1d |
| Clu6 | 5.1bc | 4.3b |
| Clu7 | 4.6c | 3.7c |
| Clu8 | 5.8ab | 4.8b |
| P level | 1.06E-12 | 6.57E-15 |
Clu3, 1.6*
Clu1, 0.9**
Clu6, 0.7**
Clu4, 0.7**
Clu7, 0.7***
Clu8, 0.7**
Clu2, 0.7*
Clu5, 0.4*

## Slide 3
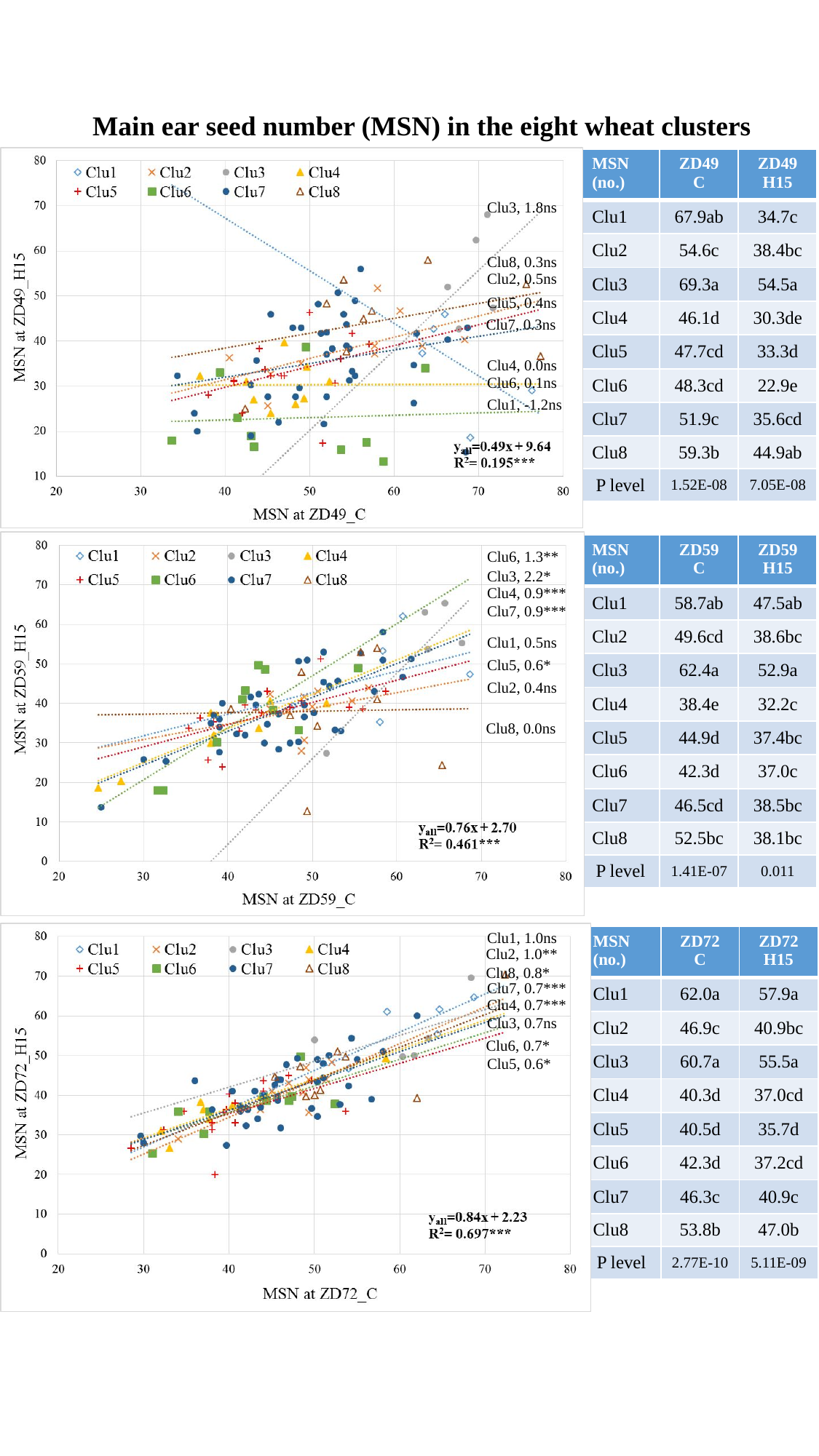

Main ear seed number (MSN) in the eight wheat clusters
| MSN (no.) | ZD49 C | ZD49 H15 |
| --- | --- | --- |
| Clu1 | 67.9ab | 34.7c |
| Clu2 | 54.6c | 38.4bc |
| Clu3 | 69.3a | 54.5a |
| Clu4 | 46.1d | 30.3de |
| Clu5 | 47.7cd | 33.3d |
| Clu6 | 48.3cd | 22.9e |
| Clu7 | 51.9c | 35.6cd |
| Clu8 | 59.3b | 44.9ab |
| P level | 1.52E-08 | 7.05E-08 |
Clu3, 1.8ns
Clu8, 0.3ns
Clu2, 0.5ns
Clu5, 0.4ns
Clu7, 0.3ns
Clu4, 0.0ns
Clu6, 0.1ns
Clu1, -1.2ns
| MSN (no.) | ZD59 C | ZD59 H15 |
| --- | --- | --- |
| Clu1 | 58.7ab | 47.5ab |
| Clu2 | 49.6cd | 38.6bc |
| Clu3 | 62.4a | 52.9a |
| Clu4 | 38.4e | 32.2c |
| Clu5 | 44.9d | 37.4bc |
| Clu6 | 42.3d | 37.0c |
| Clu7 | 46.5cd | 38.5bc |
| Clu8 | 52.5bc | 38.1bc |
| P level | 1.41E-07 | 0.011 |
Clu6, 1.3**
Clu3, 2.2*
Clu4, 0.9***
Clu7, 0.9***
Clu1, 0.5ns
Clu5, 0.6*
Clu2, 0.4ns
Clu8, 0.0ns
Clu1, 1.0ns
| MSN (no.) | ZD72 C | ZD72 H15 |
| --- | --- | --- |
| Clu1 | 62.0a | 57.9a |
| Clu2 | 46.9c | 40.9bc |
| Clu3 | 60.7a | 55.5a |
| Clu4 | 40.3d | 37.0cd |
| Clu5 | 40.5d | 35.7d |
| Clu6 | 42.3d | 37.2cd |
| Clu7 | 46.3c | 40.9c |
| Clu8 | 53.8b | 47.0b |
| P level | 2.77E-10 | 5.11E-09 |
Clu2, 1.0**
Clu8, 0.8*
Clu7, 0.7***
Clu4, 0.7***
Clu3, 0.7ns
Clu6, 0.7*
Clu5, 0.6*

## Slide 4
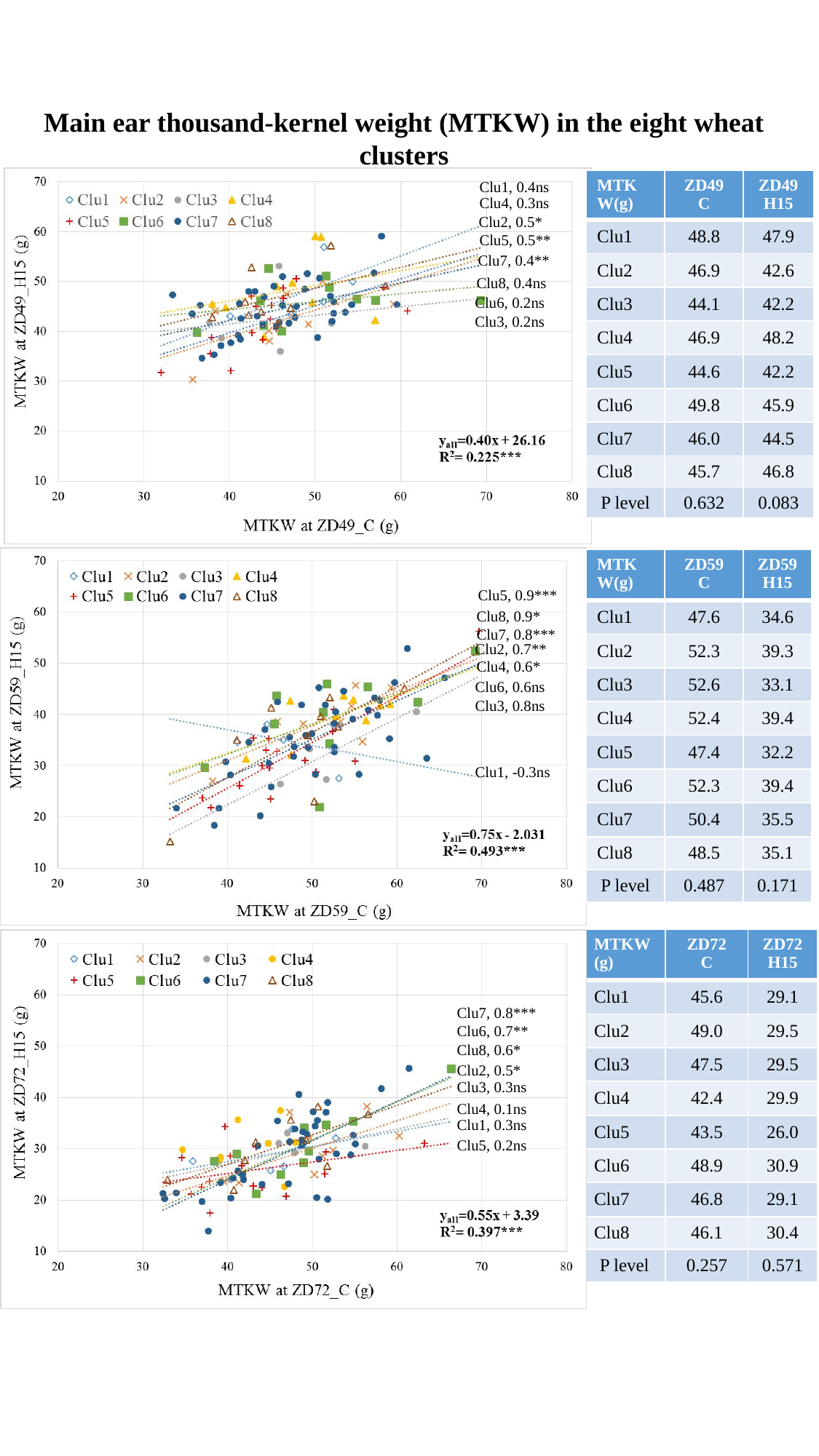

Main ear thousand-kernel weight (MTKW) in the eight wheat clusters
| MTKW(g) | ZD49 C | ZD49 H15 |
| --- | --- | --- |
| Clu1 | 48.8 | 47.9 |
| Clu2 | 46.9 | 42.6 |
| Clu3 | 44.1 | 42.2 |
| Clu4 | 46.9 | 48.2 |
| Clu5 | 44.6 | 42.2 |
| Clu6 | 49.8 | 45.9 |
| Clu7 | 46.0 | 44.5 |
| Clu8 | 45.7 | 46.8 |
| P level | 0.632 | 0.083 |
Clu1, 0.4ns
Clu4, 0.3ns
Clu2, 0.5*
Clu5, 0.5**
Clu7, 0.4**
Clu8, 0.4ns
Clu6, 0.2ns
Clu3, 0.2ns
| MTKW(g) | ZD59 C | ZD59 H15 |
| --- | --- | --- |
| Clu1 | 47.6 | 34.6 |
| Clu2 | 52.3 | 39.3 |
| Clu3 | 52.6 | 33.1 |
| Clu4 | 52.4 | 39.4 |
| Clu5 | 47.4 | 32.2 |
| Clu6 | 52.3 | 39.4 |
| Clu7 | 50.4 | 35.5 |
| Clu8 | 48.5 | 35.1 |
| P level | 0.487 | 0.171 |
Clu5, 0.9***
Clu8, 0.9*
Clu7, 0.8***
Clu2, 0.7**
Clu4, 0.6*
Clu6, 0.6ns
Clu3, 0.8ns
Clu1, -0.3ns
| MTKW (g) | ZD72 C | ZD72 H15 |
| --- | --- | --- |
| Clu1 | 45.6 | 29.1 |
| Clu2 | 49.0 | 29.5 |
| Clu3 | 47.5 | 29.5 |
| Clu4 | 42.4 | 29.9 |
| Clu5 | 43.5 | 26.0 |
| Clu6 | 48.9 | 30.9 |
| Clu7 | 46.8 | 29.1 |
| Clu8 | 46.1 | 30.4 |
| P level | 0.257 | 0.571 |
Clu7, 0.8***
Clu6, 0.7**
Clu8, 0.6*
Clu2, 0.5*
Clu3, 0.3ns
Clu4, 0.1ns
Clu1, 0.3ns
Clu5, 0.2ns

## Slide 5
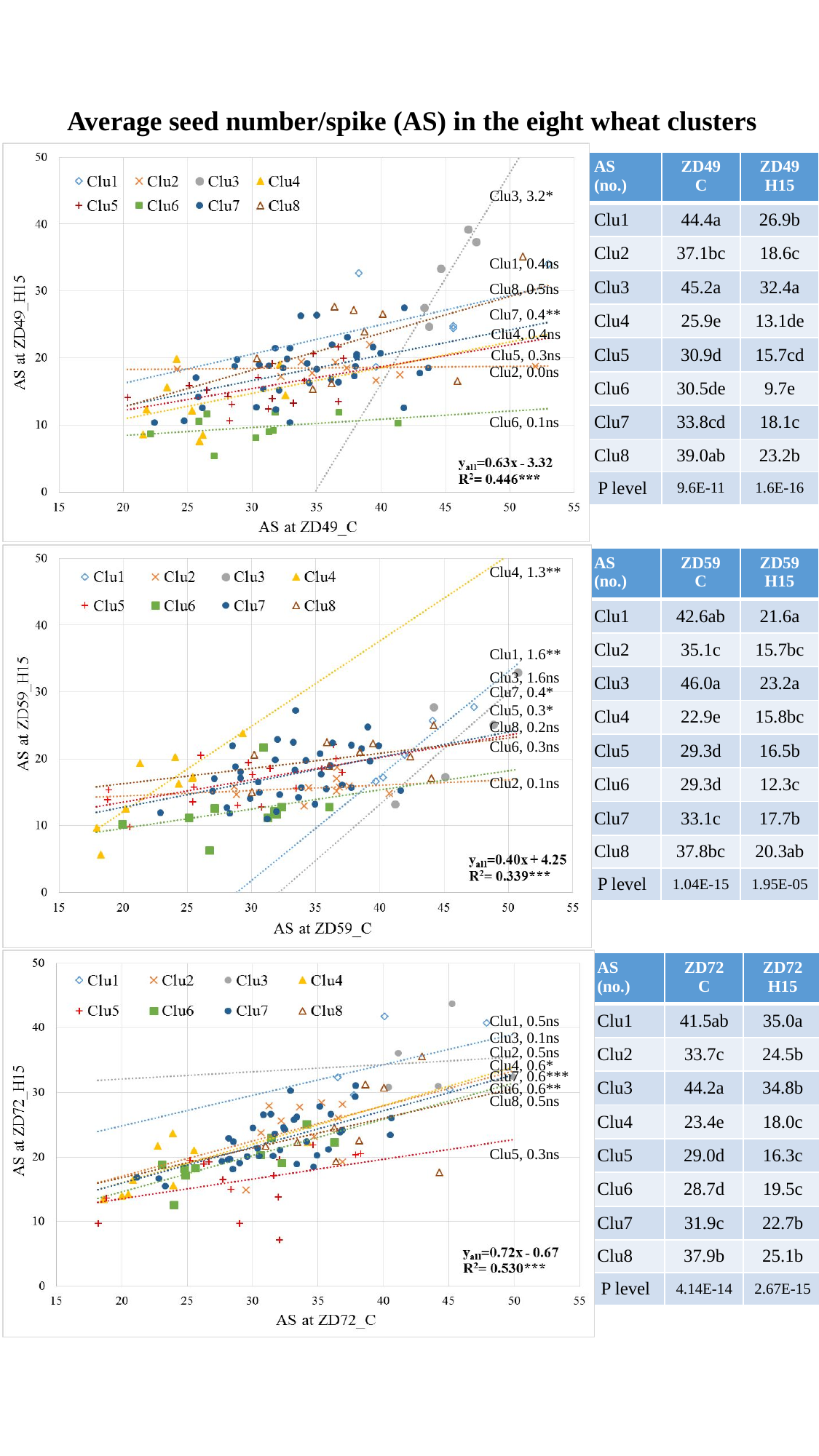

Average seed number/spike (AS) in the eight wheat clusters
| AS (no.) | ZD49 C | ZD49 H15 |
| --- | --- | --- |
| Clu1 | 44.4a | 26.9b |
| Clu2 | 37.1bc | 18.6c |
| Clu3 | 45.2a | 32.4a |
| Clu4 | 25.9e | 13.1de |
| Clu5 | 30.9d | 15.7cd |
| Clu6 | 30.5de | 9.7e |
| Clu7 | 33.8cd | 18.1c |
| Clu8 | 39.0ab | 23.2b |
| P level | 9.6E-11 | 1.6E-16 |
Clu3, 3.2*
Clu1, 0.4ns
Clu8, 0.5ns
Clu7, 0.4**
Clu4, 0.4ns
Clu5, 0.3ns
Clu2, 0.0ns
Clu6, 0.1ns
| AS (no.) | ZD59 C | ZD59 H15 |
| --- | --- | --- |
| Clu1 | 42.6ab | 21.6a |
| Clu2 | 35.1c | 15.7bc |
| Clu3 | 46.0a | 23.2a |
| Clu4 | 22.9e | 15.8bc |
| Clu5 | 29.3d | 16.5b |
| Clu6 | 29.3d | 12.3c |
| Clu7 | 33.1c | 17.7b |
| Clu8 | 37.8bc | 20.3ab |
| P level | 1.04E-15 | 1.95E-05 |
Clu4, 1.3**
Clu1, 1.6**
Clu3, 1.6ns
Clu7, 0.4*
Clu5, 0.3*
Clu8, 0.2ns
Clu6, 0.3ns
Clu2, 0.1ns
| AS (no.) | ZD72 C | ZD72 H15 |
| --- | --- | --- |
| Clu1 | 41.5ab | 35.0a |
| Clu2 | 33.7c | 24.5b |
| Clu3 | 44.2a | 34.8b |
| Clu4 | 23.4e | 18.0c |
| Clu5 | 29.0d | 16.3c |
| Clu6 | 28.7d | 19.5c |
| Clu7 | 31.9c | 22.7b |
| Clu8 | 37.9b | 25.1b |
| P level | 4.14E-14 | 2.67E-15 |
Clu1, 0.5ns
Clu3, 0.1ns
Clu2, 0.5ns
Clu4, 0.6*
Clu7, 0.6***
Clu6, 0.6**
Clu8, 0.5ns
Clu5, 0.3ns

## Slide 6
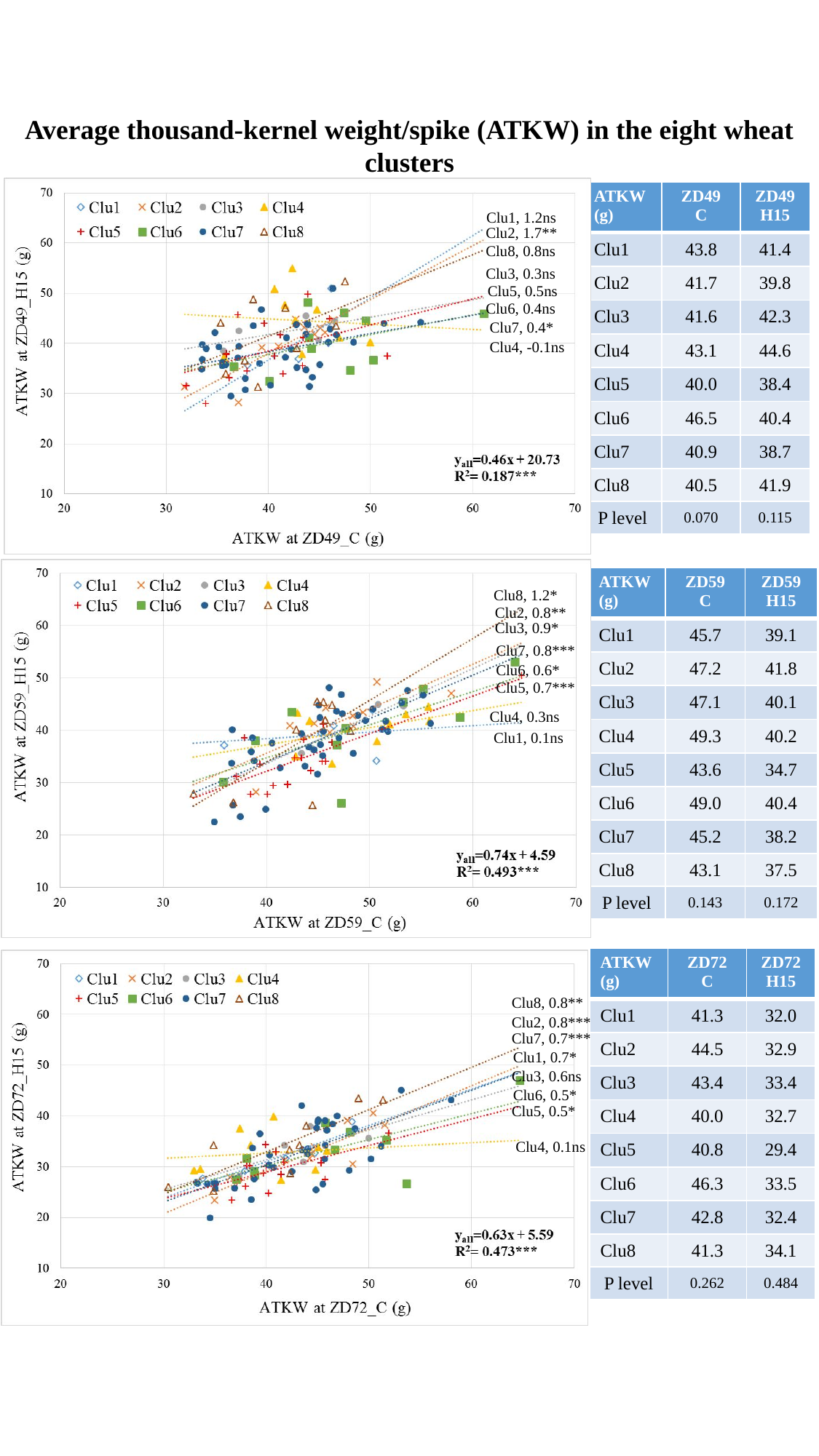

Average thousand-kernel weight/spike (ATKW) in the eight wheat clusters
| ATKW (g) | ZD49 C | ZD49 H15 |
| --- | --- | --- |
| Clu1 | 43.8 | 41.4 |
| Clu2 | 41.7 | 39.8 |
| Clu3 | 41.6 | 42.3 |
| Clu4 | 43.1 | 44.6 |
| Clu5 | 40.0 | 38.4 |
| Clu6 | 46.5 | 40.4 |
| Clu7 | 40.9 | 38.7 |
| Clu8 | 40.5 | 41.9 |
| P level | 0.070 | 0.115 |
Clu1, 1.2ns
Clu2, 1.7**
Clu8, 0.8ns
Clu3, 0.3ns
Clu5, 0.5ns
Clu6, 0.4ns
Clu7, 0.4*
Clu4, -0.1ns
| ATKW (g) | ZD59 C | ZD59 H15 |
| --- | --- | --- |
| Clu1 | 45.7 | 39.1 |
| Clu2 | 47.2 | 41.8 |
| Clu3 | 47.1 | 40.1 |
| Clu4 | 49.3 | 40.2 |
| Clu5 | 43.6 | 34.7 |
| Clu6 | 49.0 | 40.4 |
| Clu7 | 45.2 | 38.2 |
| Clu8 | 43.1 | 37.5 |
| P level | 0.143 | 0.172 |
Clu8, 1.2*
Clu2, 0.8**
Clu3, 0.9*
Clu7, 0.8***
Clu6, 0.6*
Clu5, 0.7***
Clu4, 0.3ns
Clu1, 0.1ns
| ATKW (g) | ZD72 C | ZD72 H15 |
| --- | --- | --- |
| Clu1 | 41.3 | 32.0 |
| Clu2 | 44.5 | 32.9 |
| Clu3 | 43.4 | 33.4 |
| Clu4 | 40.0 | 32.7 |
| Clu5 | 40.8 | 29.4 |
| Clu6 | 46.3 | 33.5 |
| Clu7 | 42.8 | 32.4 |
| Clu8 | 41.3 | 34.1 |
| P level | 0.262 | 0.484 |
Clu8, 0.8**
Clu2, 0.8***
Clu7, 0.7***
Clu1, 0.7*
Clu3, 0.6ns
Clu6, 0.5*
Clu5, 0.5*
Clu4, 0.1ns
